# Ionic Liquid-Based Low-Temperature Synthesis of Crystalline Ti(OH)OF·0.66H_2_O: Elucidating the Molecular Reaction Steps by NMR Spectroscopy and Theoretical Studies

**DOI:** 10.1021/acsomega.1c06534

**Published:** 2022-02-02

**Authors:** Melanie Sieland, Manuel Schenker, Lars Esser, Barbara Kirchner, Bernd M. Smarsly

**Affiliations:** † Institute of Physical Chemistry, Justus Liebig University, Heinrich-Buff-Ring 17, D-35392 Giessen, Germany; ‡ Mulliken Center for Theoretical Chemistry, Institut für Physikalische und Theoretische Chemie, Rheinische Friedrich-Wilhelms-Universität Bonn, Beringstrasse 4+6, D-53115 Bonn, Germany; § Center of Materials Research, Justus Liebig University, Heinrich-Buff-Ring 16, D-35392 Giessen, Germany

## Abstract

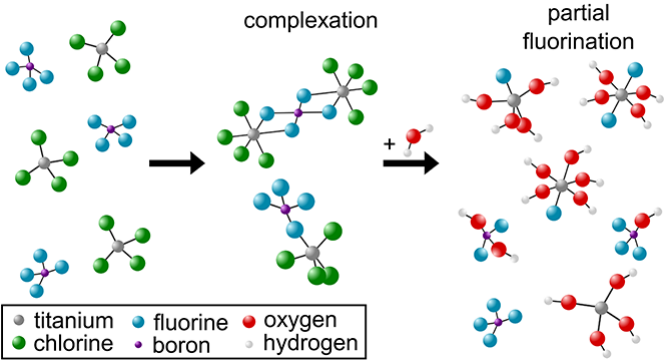

We present an in-depth mechanistic study of the first steps of the solution-based synthesis of the peculiar hexagonal tungsten bronze-type Ti(OH)OF·0.66H_2_O solid, using NMR analyses (^1^H, ^13^C, ^19^F, and ^11^B) as well as modeling based on density functional theory (DFT) and ab initio molecular dynamics (AIMD) simulation. The reaction uses an imidazolium-based ionic liquid (IL, e.g., C*_x_*mim BF_4_) as a solvent and reaction partner. It is puzzling, as the fluorine-rich crystalline solid is obtained in a “beaker chemistry” procedure, starting from simple compounds forming a stable solution (BF_4_^–^-containing IL, TiCl_4_, H_2_O) at room temperature, and a remarkably low reaction temperature (95 °C) is sufficient. Building on NMR experiments and modeling, we are able to provide a consistent explanation of the peculiar features of the synthesis: evidently, the hydrolysis of the IL anion BF_4_^–^ is a crucial step since the latter provides fluoride anions, which are incorporated into the crystal structure. Contrary to expectations, BF_4_^–^ does not hydrolyze in water at room temperature but interacts with TiCl_4_, possibly forming a TiCl_4_ complex with one or two coordinated BF_4_^–^ units. This interaction also prevents the heavy hydrolysis reaction of TiCl_4_ with H_2_O but—on the other side—spurs the hydrolysis of BF_4_^–^ already at room temperature, releasing fluoride and building F-containing Ti(OH)_*x*_Cl_4–*x*_F_*y*_ complexes. The possible complexes formed were analyzed using DFT calculations with suitable functionals and basis sets. We show in addition that these complexes are also formed using other titanium precursors. As a further major finding, the heating step (95 °C) is only needed for the condensation of the Ti(OH)_*x*_Cl_4–*x*_F_*y*_ complexes to form the desired solid product but not for the hydrolysis of BF_4_^–^. Our study provides ample justification to state a “special IL effect”, as the liquid state, together with a stable solution, the ionic nature, and the resulting deactivation of H_2_O are key requirements for this synthesis.

## Introduction

Syntheses of metal oxides involving ionic liquids (ILs) have gained substantial interest recently since it is possible to synthesize many different metal oxides with various morphologies.^1−3^ In comparison to other types of metal oxide syntheses, IL-based strategies often enable to use lower reaction temperatures. Hence, in this respect, IL-based syntheses of metal oxides are potentially consistent with the concept of sustainable chemistry.^4,5^ Interestingly, it was found that ILs possibly are not only solvents in such reactions, but they also act as reactants and thereby strongly direct the compounds obtained. This property was, for example, demonstrated in the phase-pure synthesis of the uncommon, bronze-type compound “TiO_2_(B)” with the help of imidazolium-based ILs.^6,7^ Compared to other literature-known syntheses of this compound, quite “soft” conditions, requiring a temperature of only 95 °C, are sufficient.^8^ Another major advantage is the small number of reactants, as only ILs, TiCl_4_, H_2_O, and EtOH are needed. As a crucial step of this synthesis, the usage of a mixture of two different ILs, C_16_mim Cl and C_4_mim BF_4_, was proposed. Voepel et al. further investigated the reaction mechanism of this synthesis and found that the concentration of BF_4_^–^ significantly influences the product composition.^9^ It was proposed that BF_4_^–^ is partly hydrolyzed during the reaction, providing fluoride anions, which can coordinate to Ti chloro complexes. The finally obtained bronze-type TiO_2_(B) material is not phase-pure but contains a low fraction of fluorine, which substitutes oxygen positions and thereby directs the crystallization. Depending on the amount of available fluoride anions, different amounts of blocked positions for the hydrolysis of the titanium complexes can be obtained, which for higher fractions of fluorine can even result in the formation of different titanium oxyfluoride compounds. Hence, using the synthesis of Voepel et al. beyond a certain concentration of the BF_4_-containing ILs, the peculiar hexagonal tungsten bronze (HTB) compound Ti(OH)OF·0.66H_2_O was observed,^9^ which before had been accessible by a different synthetic concept.^10^ Ti(OH)OF·0.66H_2_O possesses a quite interesting crystal structure with channels along the c-axis, endowing the compound with interesting electrochemical properties with respect to the incorporation of Li^+^ probably in the channels.^11^

In addition to the influence of the IL anion, the length of the alkyl chain of imidazolium-based ILs influences the obtained products as well,^12^ allowing us to use just one BF_4_^–^-containing IL to synthesize Ti(OH)OF·0.66H_2_O instead of a mixture of ILs, contrary to previous studies.^9^ Furthermore, within or after the formation of Ti(OH)OF·0.66H_2_O nanoparticles, the polar imidazolium head group attaches on the nanoparticles’ surface. It is probably this attachment that stabilizes the nanoparticles against conversion into the thermodynamically more stable polymorphs TiO_2_(B) and anatase. This stabilization effect is more pronounced when using ILs with longer alkyl chains demonstrating how essential the choice of the IL cation is.^12^

While thus already several details of the reaction mechanism of the presented IL-based synthesis leading to Ti(OH)OF·0.66H_2_O and TiO_2_(B) have been clarified, the first steps in the reaction, i.e., involving molecular species, are still unclear. However, the described empirical findings suggest that the final crystal structure is already predetermined at the level of F- and O-containing Ti complexes. Hence, in the present study, we target the initial reaction steps, involving Ti complexes and especially the BF_4_^–^ anion with the help of NMR spectroscopy.

In the past, NMR spectroscopy has already been successfully used to investigate IL-based syntheses.^13^ Saihara et al. investigated the hydrolysis process of the IL *N*,*N*-diethyl-*N*-methyl-*N*-(2-methoxyethyl)ammonium tetrafluoroborate (DEME BF_4_^–^) in the presence of water by means of ^19^F and ^11^B NMR measurements.^14^ They found that during the hydrolysis of BF_4_^–^, HF is generated and reacts with the surrounding glass container since SiF_6_^2–^ was detected in the ^19^F NMR spectrum. Lin et al. focused their work on anion-exchange reactions in imidazolium-based ILs leading to the formation of hydroxometalates.^15^ Since imidazolium-based ILs are also used in the already presented synthesis of Ti(OH)OF·0.66H_2_O (HTB), it can be expected that liquid-state NMR measurements can contribute to elucidate the underlying initial reaction steps. In addition, it was demonstrated by Giernoth et al. that different interactions in imidazolium-based ILs are detectable by NMR.^16^

**Scheme 1:**
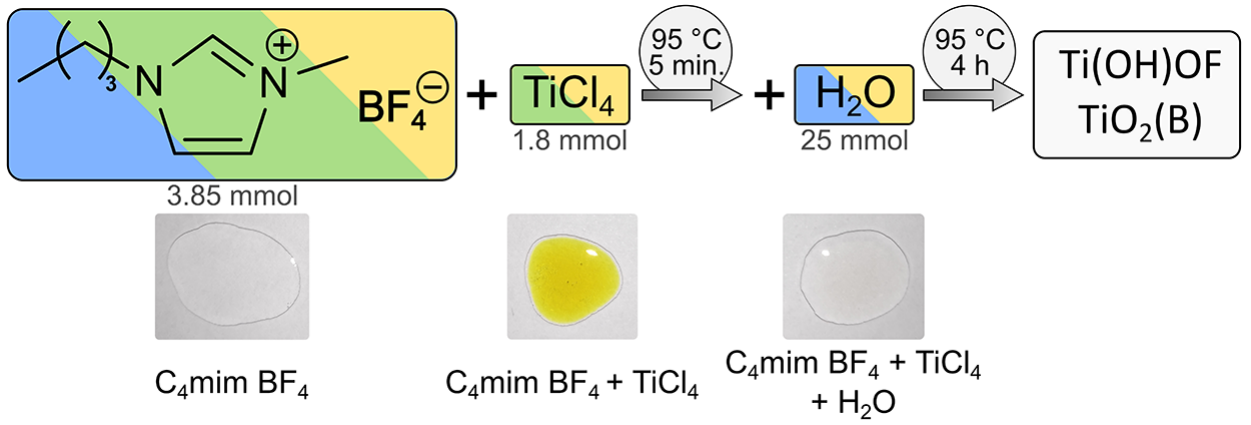
Schematic Illustration of the Synthesis Procedure The colors inside the boxes refer to the different mixtures of reactants, which were investigated in this work (blue: IL + H_2_O, green: IL + TiCl_4_, yellow: IL + TiCl_4_ + H_2_O). The shown images of the solutions correspond to the subsequent reaction steps at room temperature.

Our previous studies have already shown that the hydrolysis of the IL anion BF_4_^–^ is a crucial step, as it provides the fluoride anions that are incorporated into the crystal structure of Ti(OH)OF·0.66H_2_O. Theoretical calculations and Raman spectroscopy measurements performed in previous studies of our working group^9^ indicated that BF_4_^–^ is stepwise hydrolyzed to B(OH)_4_^–^. However, it has not been clarified yet whether the hydrolysis occurs via a direct reaction between BF_4_^–^ and H_2_O or via an interaction/reaction between BF_4_^–^ and a Ti complex, e.g., Ti(H_2_O)_*y*_(OH)_6–*y*_^*y*–2^. Hence, in the present study, we conducted ^1^H, ^13^C, ^19^F, and ^11^B NMR measurements on solutions containing different mixtures of the used reactants with the goal to elucidate intermediate products and interactions present in the solutions, to shed further light on the hydrolysis mechanism of BF_4_^–^ in the mixture with Ti complexes. Since the heating step is crucial for the synthesis of Ti(OH)OF·0.66H_2_O, additional NMR measurements were performed on solutions heated in situ to 95 °C (standard reaction conditions, see Scheme 1).

To understand the role of the Ti compound, we also used titanium isopropoxide (TTIP) for the synthesis of Ti(OH)OF·0.66H_2_O. TTIP was chosen, as here Ti is bonded to alkoxy groups, thus possessing a different hydrolysis behavior as TiCl_4_, which was previously used.

To determine if complexes between TiCl_4_ and BF_4_^–^ are present during the reaction, theoretical calculations were carried out. For this purpose, possible complexes that could form (based on the given reactants) were postulated. In the next step, these complexes, as well as their starting materials, were post-modeled and then geometry-optimized via density functional theory (DFT) calculations (see the Computational Details: Static Calculations section).

In summary, the conceptual methodology of this study is to elucidate the first reaction steps and species in the IL-mediated formation of the inorganic solid Ti(OH)OF·0.66H_2_O, to clarify how the generation of such F- and O-containing crystal is possibly predetermined at the level of molecular species, taking advantage of NMR spectroscopy and state-of-the-art modeling.

## Results and Discussion

The main goal of our investigations was to get insight into the reaction mechanisms of the IL-based synthesis of Ti(OH)OF·0.66H_2_O, especially the role of the BF_4_^–^ anion and the formation of Ti complexes, on the basis of NMR spectra. To understand the interactions of the reactants with each other, the composition of mixtures of the involved compounds was systematically varied, and these solutions were subjected to ^1^H, ^13^C, ^19^F, and ^11^B NMR measurements. A scheme showing all measured solutions and the questions we tried to answer with different NMR measurements can be found in the Supporting Information (SI) (see Scheme S1). Generally, in boron NMR, the ^11^B nucleus is used because its sensitivity is higher than that of ^10^B. Note that NMR using the Ti nucleus inherently cannot provide relevant information, as ^47^Ti and ^49^Ti possess a low sensitivity and as only a symmetric environment provides distinct signals.

### Investigation of Pure C_4_mim BF_4_

The special part of our presented synthesis is the use of imidazolium-type ILs. In our previous works, we proved that the ILs act not only as a solvent but also play a crucial role in the formation of Ti(OH)OF·0.66H_2_O, as they provide fluorine, which is integrated into the HTB compound. Since evidently BF_4_^–^ has to be hydrolyzed during the reaction, the IL anion acts as a reactant.^11,12^

**Figure 1:**
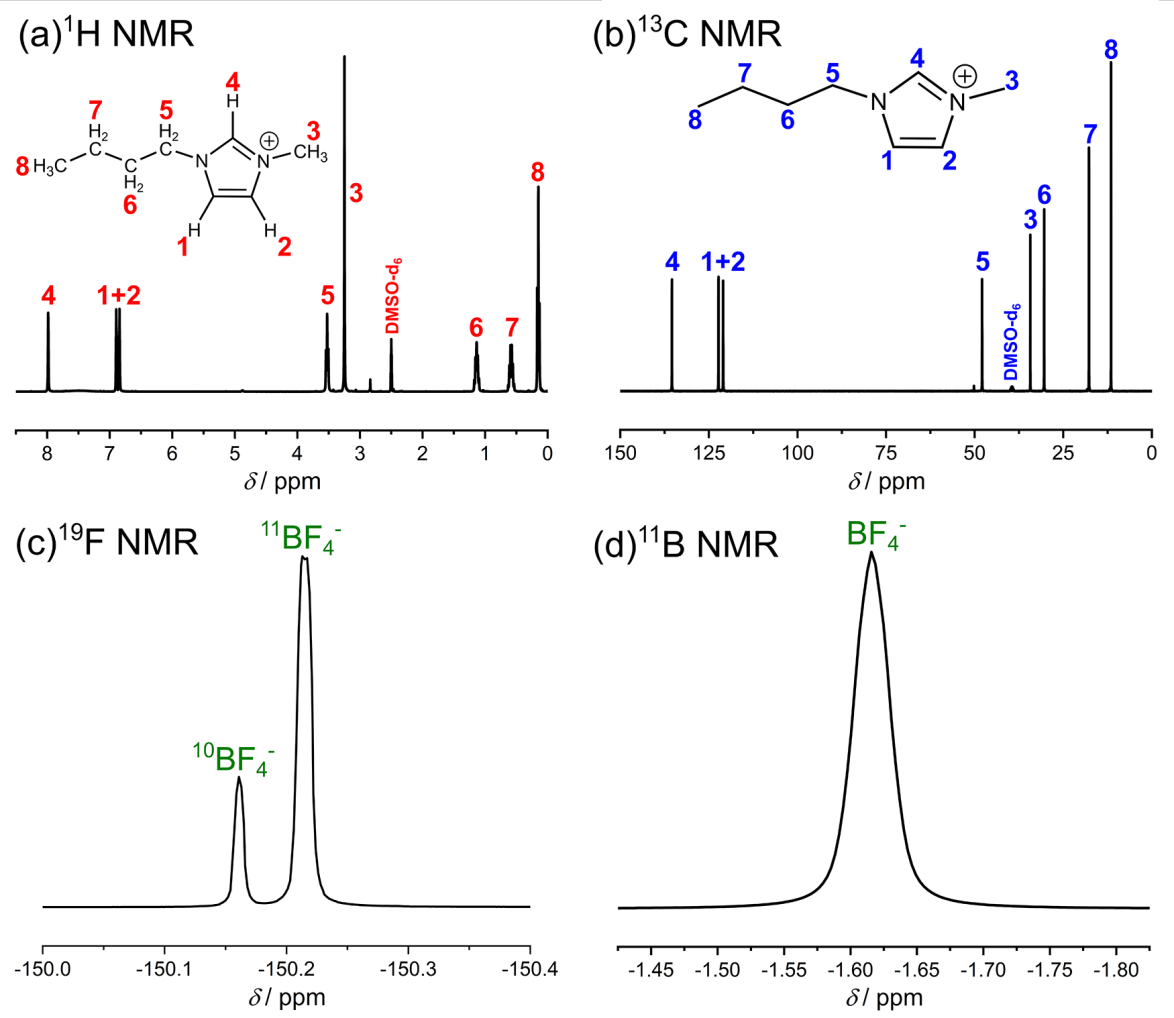
(a) ^1^H NMR (b) ^13^C NMR (c) ^19^F NMR, and (d) ^11^B NMR spectrum of pure C_4_mim BF_4_. All spectra were measured with 400 MHz at 298 K, and a solution containing 0.1 M trifluoroacetic acid (TFA) in dimethyl sulfoxide (DMSO)-*d*_6_ was used as external standard. For ^11^B NMR measurements, boron trifluoride etherate was used as a reference.

Prior to interpreting the NMR spectra of mixtures, it is illustrative to discuss the NMR patterns of the pure compounds, for comparison. Figure 1 shows the measured NMR spectra of pure C_4_mim BF_4_. The ^1^H NMR and ^13^C NMR spectra (Figure 1a,b) correspond to the structure of the IL cation, while the ^11^B NMR and ^19^F NMR spectra (Figure 1c,d) of C_4_mim BF_4_ show the typical signals of BF_4_^–^. It is noticeable that in the ^19^F NMR spectrum (Figure 1c) an isotopic chemical shift of the BF_4_^–^ peak can be observed, which is caused by the two boron isotopes ^10^B and ^11^B. The calculated integrals of the two peaks indicate that these isotopes are present in a ratio of 1 (^11^B):0.25 (^10^B), which corresponds to the natural occurrence of the isotopes and therefore proves that the different chemical shifts are caused by these isotopes.^14,25^ The ^11^B NMR spectrum (see Figure 1d) shows only one peak, which is quite broad due to the nuclear quadrupole moment of ^11^B.^26^

### Interaction of the IL C_4_mim BF_4_ with Water

The first interaction we focus on is the interaction of C_4_mim BF_4_ with H_2_O. Therefore, we prepared a solution containing 3.85 mmol C_4_mim BF_4_ and 25 mmol H_2_O, which are the standard amounts in this synthesis (see the Experimental Section), and measured ^1^H, ^13^C, ^19^F, and ^11^B spectra of this solution. The solution was prepared and measured at room temperature.

**Figure 2:**
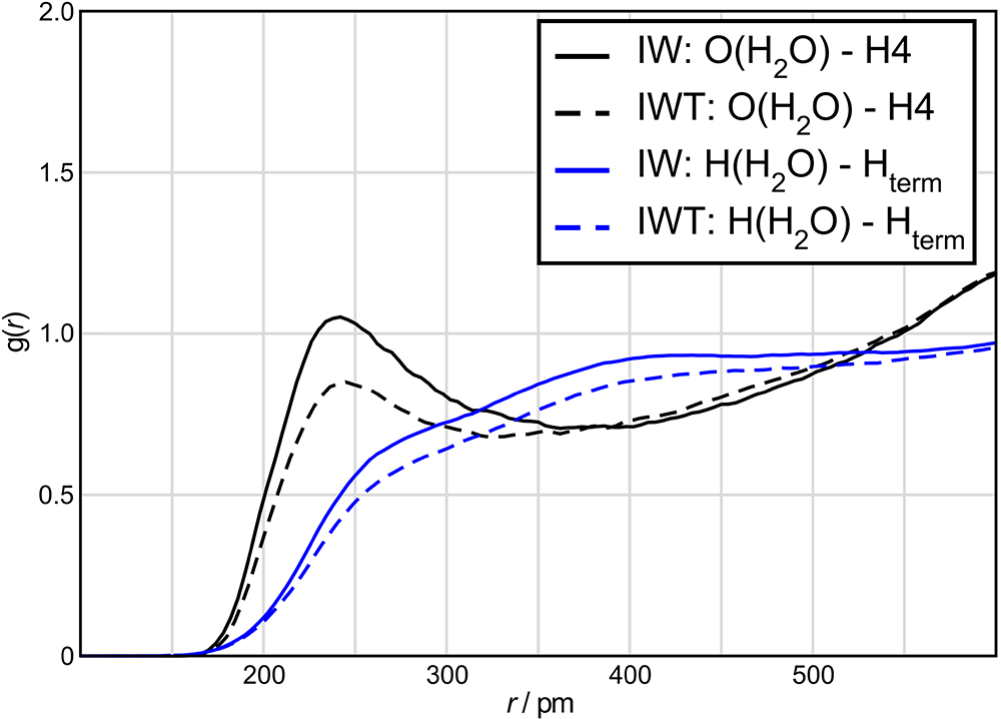
Calculated radial distribution functions (RDFs) between H(H_2_O)/O(H_2_O) and the most acidic hydrogen atom H4 and the terminal hydrogen atoms of the butyl chain H_term_. IW refers to a solution containing IL and H_2_O; IWT refers to a solution containing IL, H_2_O, and TiCl_4_. The data are adapted from ref (24).

The ^1^H and ^13^C spectra (see Figure S4) prove that the cation is not affected by the presence of water. Similar observations have been reported in the theoretical studies of similar systems, providing the radial distribution functions (RDFs) of different solutions.^24^ The RDFs calculated for a mixture of IL and water (see Figure 2) show that water interacts for the most part only with the polar components of the imidazolium cation, in this case with the most acidic hydrogen atom H4 of the imidazolium ring, which is reflected in the RDFs of O(H_2_O)-H4. In contrast, the terminal hydrogen atoms of the butyl side chain of the imidazolium cation (denoted as H_term_) are counted as nonpolar components and the corresponding RDFs between H_term_ and water show hardly any signals. The different RDFs therefore demonstrate that water only exhibits interactions with H4 and not with H_term_. Therefore, we conclude that the butyl side chain is not affected by the addition of water, which is in agreement with the already mentioned ^1^H and ^13^C NMR results.

**Figure 3:**
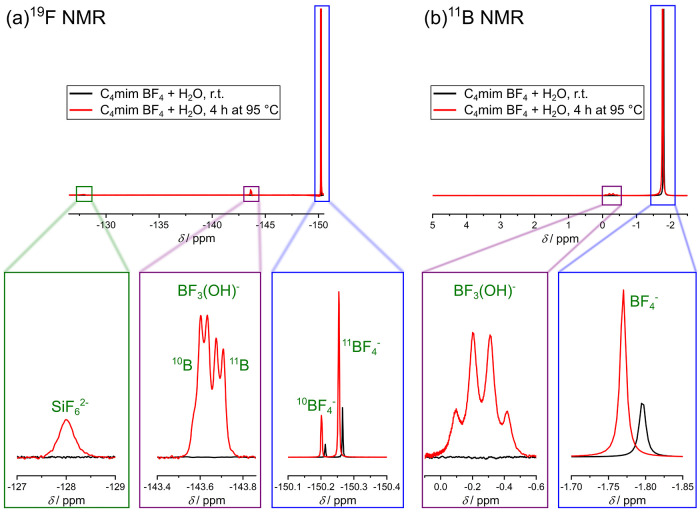
(a) ^19^F NMR and (b) ^11^B NMR spectrum of a mixture containing C_4_mim BF_4_ and H_2_O with a molar ratio of 1:6.5 which was heated to 95 °C for 4 h. All spectra were measured with 400 MHz at 298 K, and a solution containing 0.1 M TFA in DMSO-*d*_6_ was used as external standard. For ^11^B NMR measurements, boron trifluoride etherate was used as reference.

The results of the ^19^F and ^11^B NMR measurements suggest that the anion is not affected either, as in the ^19^F spectrum still the characteristic signal of BF_4_^–^ is present, with the two signals originating from isotopic chemical shift showing a ratio of 1 (^11^B):0.24 (^10^B) (see Figure 3a). It is notable that in both spectra the signals are shifted compared to the pure IL (see Figure 1). This shift can be explained by the different concentration of C_4_mim BF_4_ in the measured solutions. Since no significant changes in the spectra were observed, it can be stated that the addition of water at room temperature does not affect the IL. No hydrolysis of BF_4_^–^ seems to have taken place at the time of the measurement, although literature reports that hydrolysis of BF_4_^–^ might occur at room temperature, but it needs several days (depending on the amount of water used) to produce a detectable amount of hydrolysis products (e.g., BF_3_(OH)^−^).^14,27^ It is therefore understandable why no hydrolysis products were observed in our spectra since they were measured within a period of 12 h after mixing the IL with H_2_O.

After heating the solution for 4 h at 95 °C (typical reaction time and temperature, see the Experimental Section) and subsequent cooling to room temperature, it was possible to detect hydrolysis products of BF_4_^–^. The ^19^F NMR spectrum (see Figure 3a) contains, besides the BF_4_^–^ signals, several signals in the range of −143.5 to −143.8 ppm. Based on the characteristic splitting of the peak, which was already reported in the literature, these signals can be assigned to BF_3_(OH)^−^, a hydrolysis product of BF_4_^–^.^14^ The signal at −128.0 ppm can be attributed to SiF_6_^2–^, which is generated as a result of the formation of small amounts of HF during the hydrolysis reacting with the glass of the NMR tube. The peaks corresponding to BF_4_^–^ and BF_3_(OH)^−^ are also visible in the measured ^11^B NMR spectrum (see Figure 3b). It can therefore be concluded that a higher reaction temperature leads to a faster hydrolysis of BF_4_^–^. At the same time, the IL cation is not affected since the ^1^H and ^13^C NMR spectra of this solution (see Figure S5) are comparable to the spectra of pure C_4_mim BF_4_.

### Interaction of Different ILs with TiCl_4_

The next interaction we wanted to focus on was the interaction/reaction of C_4_mim BF_4_ with TiCl_4_. We assumed that an interaction is crucial for a successful synthesis of Ti(OH)OF·0.66H_2_O, in order to reduce the reactivity of TiCl_4_, establishing a stable solution in the first step of the synthesis (Scheme 1). Otherwise, after the addition of H_2_O to the solution an immediate and heavy reaction of TiCl_4_ would occur, resulting in the formation of other titanium oxides (e.g., anatase) instead of the uncommon HTB compound.^28^

Hence, we prepared a sample containing 3.85 mmol C_4_mim BF_4_ and 1.82 mmol TiCl_4_ (standard ratio for a reaction leading to Ti(OH)OF·0.66H_2_O)^12^ and measured different NMR spectra of this sample. The structure of the cation is not affected by the presence of TiCl_4_, since the ^1^H and ^13^C NMR spectra (see Figure S6) are comparable to the spectra of pure C_4_mim BF_4_. Interestingly the position of the peaks is shifted in both spectra. In the ^1^H NMR spectrum, all signals are shifted to higher values, with the shifts of the protons located on the imidazolium ring ranging from 0.16 to 0.18 ppm. The shifts of the protons of the alkyl side chain, on the other hand, are larger, ranging from 0.25 to 0.29 ppm. Voronoi analysis of a solution containing C_4_mim BF_4_ and TiCl_4_ performed in a previous study^24^ indicated that the reference surfaces of TiCl_4_ are largely covered by the imidazolium ring, and possible surface coverage by the alkyl chain is prevented. The differing shift ranges observed in the ^1^H NMR spectrum are therefore a result of TiCl_4_ located mostly near the imidazolium ring. The visible shifts in the ^13^C NMR spectrum support this prediction since the shifts also vary depending on the position of carbon inside of the cation.

**Figure 4:**
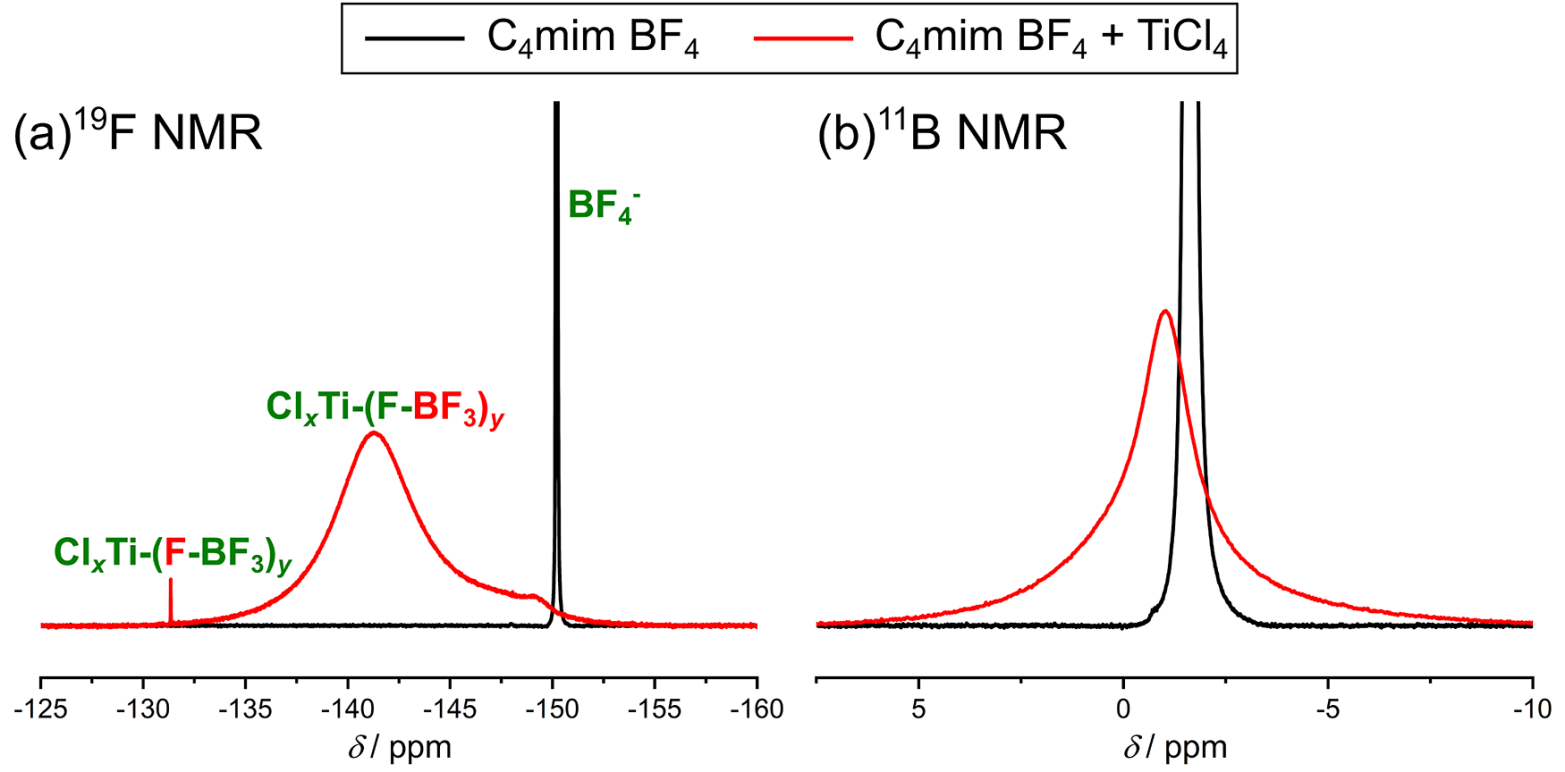
(a) ^19^F NMR and (b) ^11^B NMR spectrum of a mixture containing C_4_mim BF_4_ and TiCl_4_ in a ratio of 1:0.5. All spectra were measured with 400 MHz at 298 K, and a solution containing 0.1 M TFA in DMSO-*d*_6_ was used as external standard. For ^11^B NMR measurements, boron trifluoride etherate was used as a reference.

The ^19^F and ^11^B NMR spectra (see Figure 4) clearly show that an interaction of the BF_4_^–^ anion with TiCl_4_ must be present in this sample. The ^19^F NMR spectra (see Figure 4a) contain no longer the significant peak of BF_4_^–^observed for pure C_4_mim BF_4_. Instead, several other peaks, some of them being quite broad, can now be observed. The ^11^B NMR spectrum (see Figure 4b) of the mixture shows a quite broad signal, in comparison to the pattern of pure C_4_mim BF_4_, and the position of this peak is slightly shifted due to the different concentration of the IL and probably a different environment of the boron center. The broad signal visible in the spectrum can be explained by the different chemical environment of boron as well. Literature has shown that the line width in ^11^B NMR measurements strongly depends on the coordination and symmetry around the boron center. Moving from the highly symmetric BF_4_^–^ to a less symmetric compound increases the line width therefore we can conclude that BF_4_^–^ is no longer present, which is in agreement with the ^19^F NMR results.^29^ These observations indicate some kind of interaction between the IL anion and TiCl_4_, while at the same time no interaction between the IL cation and TiCl_4_ takes place.

Comparable observations were found in recent theoretical studies using the ab initio molecular dynamic (AIMD) simulations.^24^ In the solvation structure of TiCl_4_ in both pure and water-diluted systems, there are minor interactions between IL cations and TiCl_4_. In contrast, interactions between titanium and fluorine of tetrafluoroborate can be observed in the radial distribution functions Ti-F([BF_4_]^−^) in a mixture without water.^24^

The NMR spectra thus prove that a part of the fluorine atoms lies within a different chemical environment. Given the small number of compounds, presumably, the coordinative environment of Ti has changed. The change in coordination of Ti is further evidenced by the yellow color of the obtained solution, which is markedly more intense than pure TiCl_4_ (see Scheme 1).

**Figure 5:**
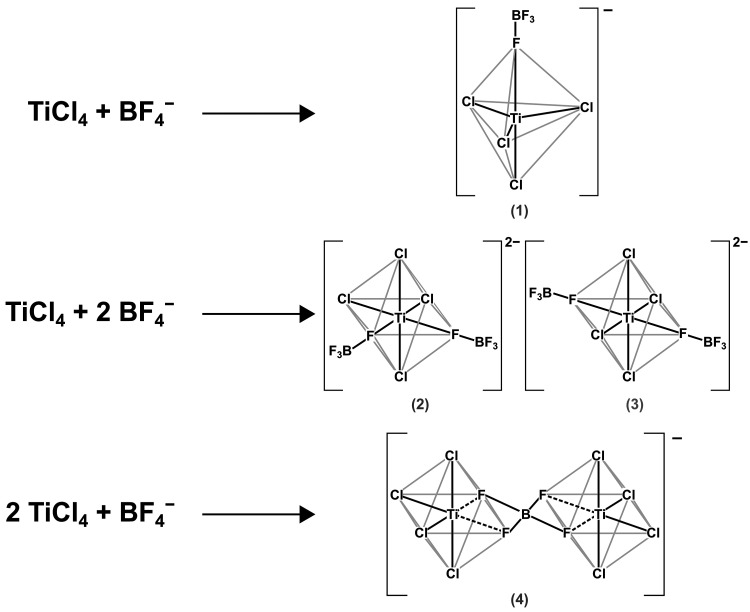
Possible complexes of TiCl_4_ and BF_4_^–^.

To elucidate the nature of such Ti complexes, we had to look closer into the measured NMR spectra. As mentioned above, there was no significant shift of the signal in the ^11^B NMR spectrum (see Figure 4b). We thus assumed that a significant portion of B–F bonds is unperturbed in this solution. It can be excluded that any compound containing B–Cl bonds was formed, as literature reports that the chemical shifts of comparable compounds are different from the observed shifts (BCl_3_: δ = 46.5 ppm; BClF_2_: δ = 20.0 ppm; BCl_2_F: δ = 31.2 ppm).^30^ With this in mind, we concluded that there are still BF_4_^–^ units present in our solution and that complexes containing TiCl_4_ and BF_4_^–^ are built. Figure 5 shows some of the possible complexes.

**Figure 6:**
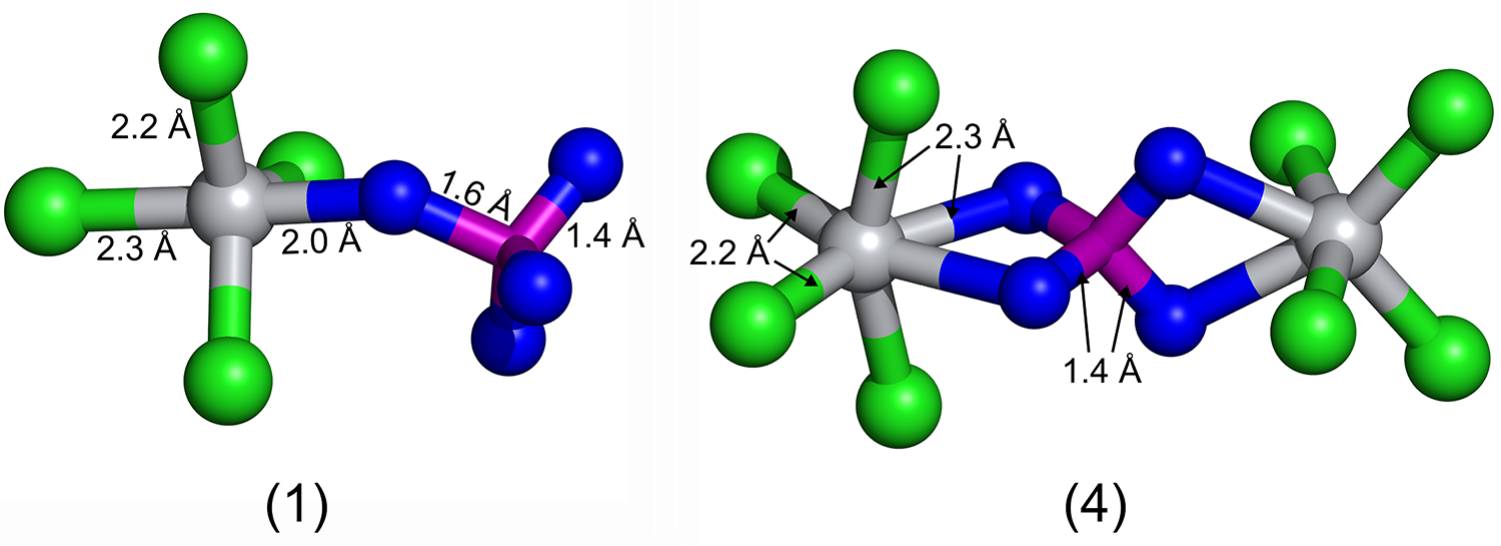
Sterical configuration of the two complexes (1) and (4) (numbering according to Figure 5), based on theoretical calculations. Titanium atoms are depicted in gray, chlorine atoms in green, fluorine atoms in blue, and boron atoms are depicted in pink.

To clarify, which complexes are possibly formed, DFT calculations were performed. In particular, for structures (1) and (4) (Figure 5), the electron, total thermal, and total enthalpic energies, as well as the Gibbs free enthalpy appear energetically favorable. Thus, according to the quantum chemical calculations, the formation of a binary TiCl_4_ complex via side-linking by tetrafluoroborate and the formation of a TiCl_4_ complex with one coordinated BF_4_^–^ unit is realistic. As an important result, these simulations suggest the complexation proceeds via a direct linkage to fluorine. By contrast, the coordination of two tetrafluoroborate units to TiCl_4_ in cis or trans configuration (structures (2) and (3)) is unlikely. The calculated electron, total thermal, and total enthalpic energies, as well as the calculated Gibbs free enthalpies are given in Table S2 in the Supporting Information. Figure 6 shows the sterical configuration of complexes (1) and (4) according to theoretical calculations. At first glance, it appears that the initial assumptions of complexes (1) and (4) (Figure 5) agree well with the geometry-optimized structures shown in Figure 6. A comparison of our calculated bond lengths (see Figure 6) with the bond lengths of literature-known compounds^31^ (TiCl_4_ (bond lengths (Ti–Cl) = 2.17–2.18 Å), TiF_4_ (bond lengths (Ti–F) = 1.75–1.77 Å) and Ti_2_F_8_ (bond lengths (Ti–F) = 1.73–1.76 Å, bond lengths (Ti–F–Ti) = 1.89–2.13 Å)) show that our calculated bond lengths are in all cases slightly larger but they are on the same order of magnitude.

The assumption that linkage of two TiCl_4_ units via BF_4_^–^ is possible can be inferred from the results in the literature^30^ as well since it is shown that for two TiF_4_ units the linkage via fluorine to form Ti_2_F_8_ dimers is possible. This shows that a linkage via F^–^, where F^–^ is connected to at least one titanium atom, is possible which is in agreement with our presented results. In contrast, the connection of two single TiCl_4_ units into a Ti_2_Cl_8_ dimer is unfavorable, in this case only weakly interacting van der Waals dimers are formed.

**Figure 7:**
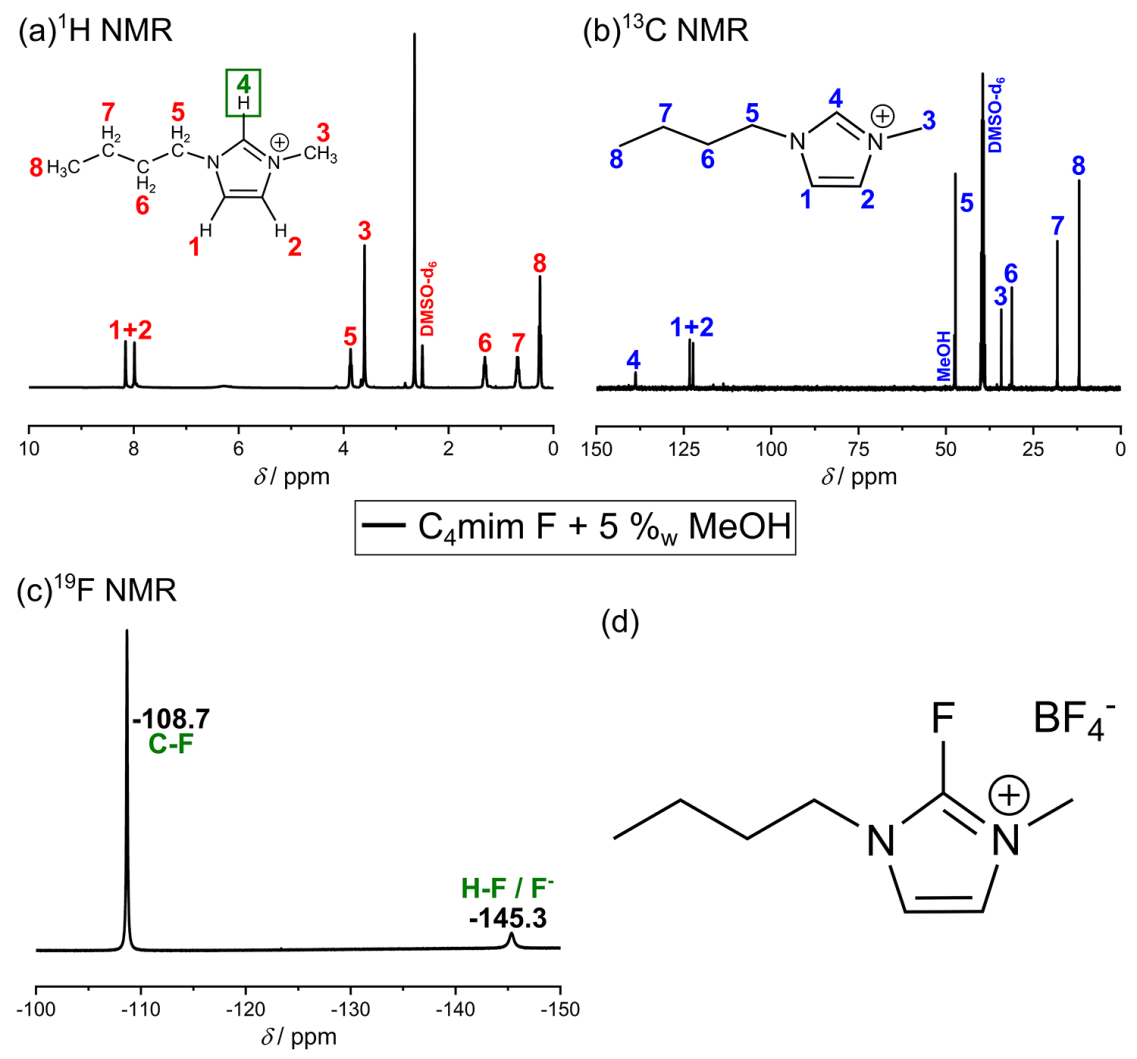
(a) ^1^H NMR, (b) ^13^C NMR, and (c) ^19^F NMR spectrum of a mixture containing C_4_mim F and 5%_w_ MeOH. All spectra were measured with 400 MHz at 298 K, and a solution containing 0.1 M TFA in DMSO-*d*_6_ was used as external standard. (d) Structural formula of the IL.

The quite broad signal in the ^19^F NMR spectrum (see Figure 4a) between −130 and −150 ppm can be attributed to a mixture of different complexes, which are, based on the theoretical calculations, complexes (1) and (4). We assume the broadness of the signal is caused by the different complexes in which different coordination environments of the fluorine are present. Interestingly, besides this broad maximum, there was an additional quite sharp signal at −131.3 ppm, which can be possibly interpreted as either isolated F^–^ or a Ti–F bond in complex (1). To clarify which of the two possibilities applies, we performed reference measurements using C_4_mim F. It should be noted that the pure compound C_4_mim F is not stable; therefore, it was necessary to use a solution of this IL containing 5%_w_ MeOH, the latter evidently impeding the interpretation of the NMR spectra. For comparison, NMR spectra of a solution containing C_4_mim F and 5%_w_ MeOH were recorded (Figure 7).

The peaks in the ^1^H NMR spectrum were comparable to the spectrum of pure C_4_mim BF_4_ (see Figure 1a), except that no signal originating from H4 was visible. However, the C4 peak was still observable in the ^13^C NMR spectrum (see Figure 7b). Consequently, an exchange reaction at C4 probably occurred in this solution (C_4_mim F and 5%_w_ MeOH), which is supported by the fact that two different peaks are visible in the ^19^F NMR spectrum (see Figure 7c). During the exchange reaction, hydride anions are released, which react with MeOH to form H_2_. Since the chemical has already been delivered as a mixture of C_4_mim F and MeOH, it was evidently impossible to observe the formation of H_2_, which would support this interpretation. With the help of literature-known compounds “AlkylFluor” (δ(C–F) = −107.51 ppm) and “PhenoFluor” (δ(C–F) = −34.15 ppm) (the structure of both compounds is shown in Figure S2), we were able to conclude that the imidazolium ring in this compound is still intact and that the peak at −108.7 ppm is related to a C–F bond at the C4 position (see Figure 7d).^17^ The second peak at −145.3 ppm can be assigned to HF/F^–^, HF being formed in the reaction of F^–^ with MeOH.^32^

**Figure 8:**
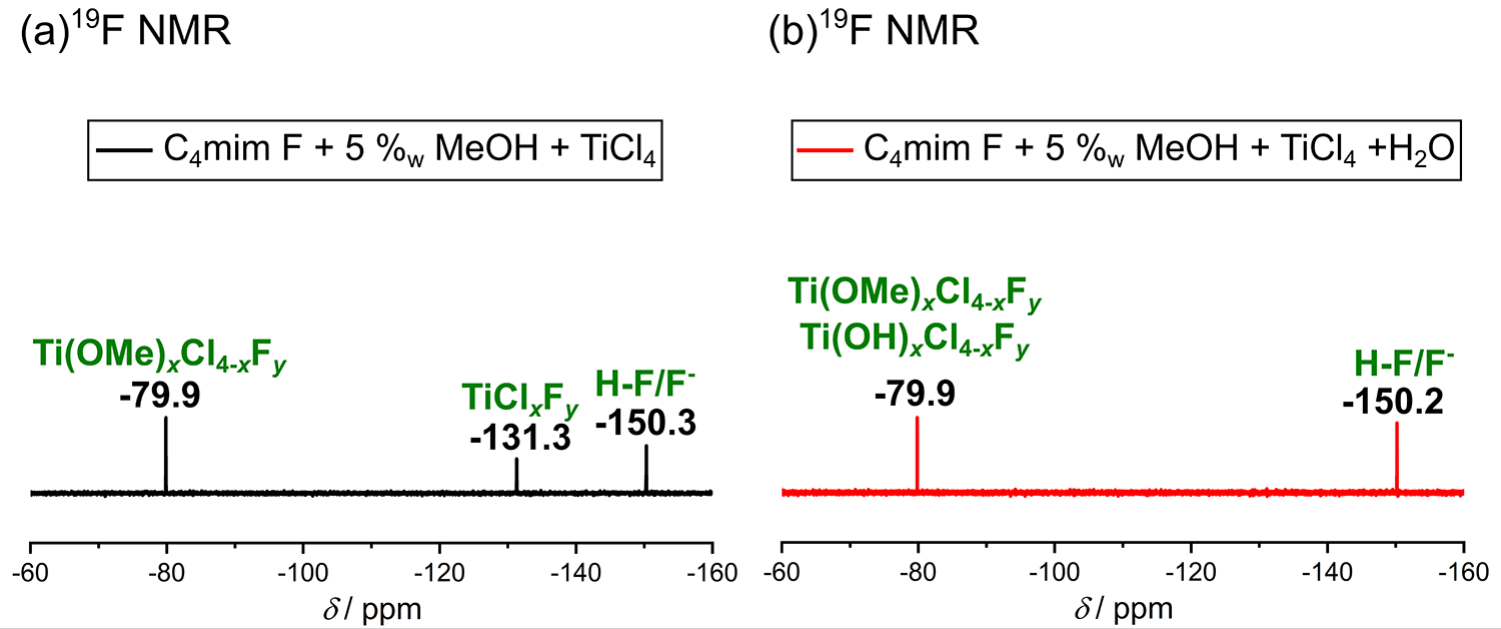
(a) ^19^F NMR spectrum of a mixture containing C_4_mim F (with 5%_w_ MeOH) and TiCl_4_ in a ratio of approximately 1:0.5. (b) ^19^F NMR spectrum of a mixture containing C_4_mim F (with 5%_w_ MeOH), TiCl_4_, and H_2_O in a ratio of approximately 1:0.5:6.9. For both spectra, the chemicals were mixed at room temperature and all spectra were measured with 400 MHz at 298 K. It was not possible to use a solution containing 0.1 M TFA in DMSO-*d*_6_ as external standard since the intensity of the ^19^F NMR signals of the solution is quite low in comparison to the peak intensity of TFA. This high intensity would mask the signals of interest.

After the addition of TiCl_4_ to the C_4_mim F-in-MeOH solution (in a molar ratio of approximately 0.5:1), a change in the measured NMR spectra can be observed. The ^1^H and ^13^C NMR spectra (see Figure S7) show that the overall structure of the cation is preserved. Interestingly, in this solution, the H4 atom is visible, which means that there is no C–F bond at position C4, which is also proven by the ^19^F NMR spectra (see Figure 8a) since the peak at −108.7 ppm is no longer visible. Instead, the ^19^F NMR spectrum exhibits three peaks with a quite low intensity. The peak at −150.3 ppm is attributable to the HF/F^–^ peak, based on a comparison with the bare C_4_mim F-in-MeOH solution (see Figure 7c). For the other two peaks (−131.3 and −79.9 ppm), we assume titanium-fluorine compounds. Comparing them with the ^19^F NMR spectrum of the C_4_mim BF_4_/TiCl_4_ mixture (see Figure 4a), interestingly the peak at −131.3 ppm occurs in both solutions. Since TiCl_4_ and an F^–^ containing IL anion are present in both solutions, this peak originates from species containing Ti–F bonds. In turn, for the synthesis using C_4_mim F a complex comparable to the complexes in Figure 5 is present, with the difference that instead of the BF_4_^–^ ligand now F^–^ is bound to the Ti atom. Also, we conclude that in the case of the synthesis applying C_4_mim BF_4_, the BF_4_^–^ unit is attached to Ti via a fluorine atom, and probably a chlorine–fluorine complex of the type TiCl_*x*_F_*y*_ is generated. This interpretation is in agreement with the already mentioned AIMD simulations, as such an interaction between fluoride and titanium was observed in the RDFs Ti-F([BF_4_]^−^) in a mixture without water.^24^

The ^19^F NMR spectrum of the C_4_mim BF_4_/TiCl_4_ mixture (see Figure 4a) shows that the bridging F^–^ atom has a different shift range (−131.3 ppm) from the other, free-moving F^–^ attached to boron (broad signals between −130 and −150 ppm) due to its bonding with Ti. In addition, this interpretation explains the sharpness of the observed peak in comparison to the other visible peaks, since there is less movement of the bridging F^–^ atom possible in comparison to the other fluorine atoms. Such different shift ranges of the bridging fluorine atom have also been observed for other metal complexes with BF_4_^–^.^33^

We assume that the peak at −79.9 ppm (Figure 8a) observed for the mixture of C_4_mim F (with 5%_w_ MeOH) and TiCl_4_ can also be attributed to a Ti–F bond with the difference that in this case a substantial fraction of chlorine is replaced by methoxy groups (−OCH_3_), generated by the reaction of TiCl_4_ with MeOH. This is supported by the fact that gas formation (HCl) was observed after the addition of TiCl_4_ to the C_4_mim F/MeOH solution. It is therefore reasonable to assume that the peak at −79.9 ppm belongs to a Ti(OMe)_*x*_Cl_4–*x*_F_*y*_ species.

Such assumption is additionally supported by the fact that after adding water in excess to the solution containing C_4_mim F, MeOH, and TiCl_4_, the peak at −131.3 ppm (see Figure 8b) disappears, while the other two peaks are still present. In this solution, due to the high amount of water, no Cl^–^-containing titanium is left, explaining the absence of the −131.3 ppm signal. Also, in the presence of water TiCl_4_ swiftly reacts with H_2_O, forming Ti–OH bonds and gaseous HCl,^9^ finally resulting in Ti(OH)_*x*_Cl_4–*x*_F_*y*_, in which fluorine experiences a similar environment as in Ti(OMe)_*x*_Cl_4–*x*_F_*y*_ species, thereby causing the signal at −79.9 ppm.

Another evidence for the ^19^F NMR signal at −79.9 ppm belonging to F^–^- and OH^–^-containing titanium complexes is its nonappearance in the C_4_mim BF_4_/TiCl_4_ solution (Figure 4a), which is understandable in the light of the absence of H_2_O or MeOH. However, the peak pops up as soon as water is added, which will be discussed in detail in the Interactions Inside of the Reaction Solution section.

### Solutions of C_4_mim BF_4_ with Titanium Isopropoxide (Ti[OCH(CH_3_)_2_]_4,_ TTIP)

Based on the finding that different complexes containing fluorine and titanium can be detected in ^19^F NMR spectra, the proposed reactions and complexes were further studied using a different Ti precursor. Ti[OCH(CH_3_)_2_]_4_ (TTIP) was chosen, as here Ti is bonded to alkoxy groups, thus resulting in a substantially different hydrolysis behavior compared to TiCl_4_. Like TiCl_4_, TTIP is not stable against hydrolysis, and TiO_2_ is built as soon as TTIP gets in contact with water. It is thus necessary to stabilize the compound, for example with the help of a conc. aqueous HCl solution, as already reported in the literature.^34^ Since a solution containing just C_4_mim BF_4_ and TTIP was not stable either, it was thus not possible to measure NMR spectra without the addition of conc. HCl_aq._, therefore all NMR measurements with TTIP contain H_2_O. Table 1 summarizes the amount of all precursors present in the two different investigated solutions with TTIP.

**Table 1:** Quantities of the Precursors in the Two Investigated Solutions Containing TTIP

solution	*n*(C_4_mim BF_4_) (mmol)	*n*(TTIP) (mmol)	*n*(HCl) (mmol)	*n*(H_2_O) (mmol)
IL + TTIP + HCl_aq_	3.85	1.70	8.09	27.89
IL + TTIP + HCl_aq_ + H_2_O	3.85	1.70	8.09	52.89

Figure S9 shows the ^1^H, ^13^C, and ^11^B NMR spectra of a solution containing C_4_mim BF_4_/TTIP/HCl/H_2_O in a molar ratio of approximately 1:0.44:2.10:7.24. The ^1^H and ^13^C NMR spectra are comparable to the spectra measured for pure C_4_mim BF_4_, indicating that the IL cation is not affected in this solution. The ^11^B NMR spectrum shows a signal at approximately −1.75 ppm, which is comparable to the observed peak for pure C_4_mim BF_4_; therefore, it can be concluded that B–F bonds are still present.

**Figure 9:**
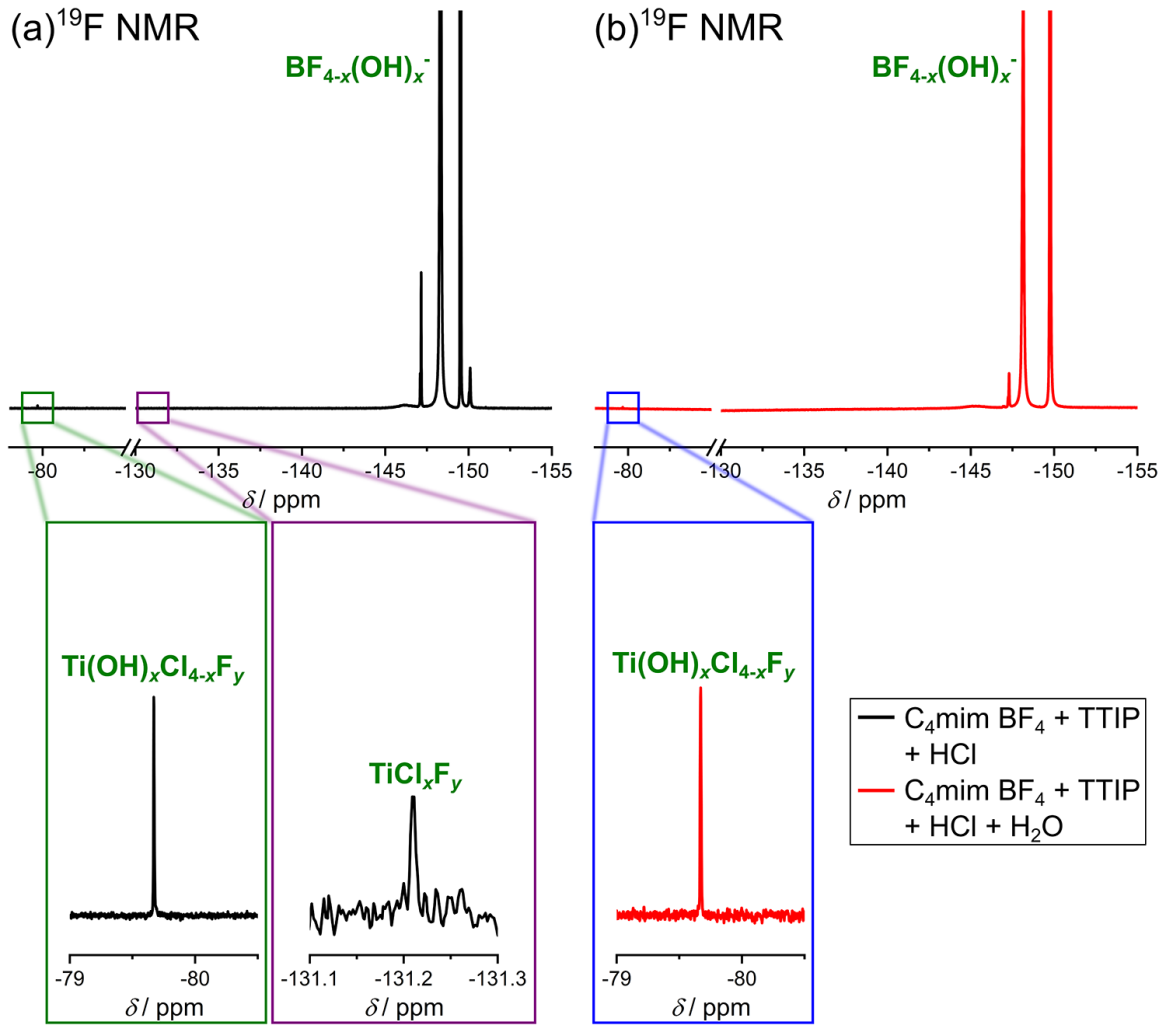
(a) ^19^F NMR spectrum of a mixture containing C_4_mim BF_4_, TTIP, and conc. HCl_aq._ in a molar ratio of approximately 1:0.44:2.10 (HCl):7.24 (H_2_O). (b) ^19^F NMR spectrum of a mixture containing C_4_mim BF_4_, TTIP, conc. HCl_aq_, and H_2_O in a molar ratio of approximately 1:0.44:2.10 (HCl):13.74 (H_2_O). All spectra were measured with 400 MHz at 298 K, and a solution containing 0.1 M TFA in DMSO-*d*_6_ was used as external standard.

In the ^19^F NMR spectrum (see Figure 9a) of the solution, it is possible to observe multiple signals in a shift range of −147 to −151 ppm. It is noticeable that all peaks with quite high intensities in this range have either a nearby, less intense peak or a shoulder. This finding indicates that these peaks can be assigned to compounds containing fluorine and boron since boron has two different isotopes, which influences the spectra (this finding was already explained in the Investigation of Pure C_4_mim BF_4_ section). The peaks at −149.46 and −149.52 ppm can, depending on their position and high intensity, be matched to BF_4_^–^. The integrals of these two peaks have a ratio of 1:0.24 being again in agreement with the natural occurrence of the two boron isotopes ^11^B and ^10^B.^14^ The other observable peak can be assigned to different hydrolysis products of BF_4_^–^ (BF_4–*x*_OH_*x*_^–^). In addition to the signals of the different B–F compounds, two other peaks are observed (−131.2 and −79.7 ppm). They are in agreement with the peaks observed in Figures 4a and 8, suggesting that comparable compounds (TiCl_*x*_F_*y*_ and Ti(OH)_*x*_Cl_4–*x*_F_*y*_) are present. Interestingly, these results prove that not all isopropoxide units at TTIP were replaced with OH^–^ units, although this would be possible by stoichiometry due to the amount of water present within the solution. Theoretical calculations have shown that large parts of H_2_O are located on the surface of the IL cation and anion in any solution containing C_4_mim BF_4_ and H_2_O.^24^ Therefore, not all H_2_O molecules are available for the hydrolysis of every TTIP unit, resulting in the presence of the NMR signal indicative of TiCl*_x_*F_*y*_ (−131.2 ppm). It should be noted that the intensity of the peak at −131.2 ppm is quite low in comparison with the peak at −131.3 ppm in Figure 8. This finding can be explained by the fact that there is more H_2_O in this solution than MeOH in the C_4_mim F + 5%_w_ MeOH + TiCl_4_ solution. As a result, more isopropoxide units are already replaced by H_2_O, which decreases the intensity of the peak at −131.2 ppm. The low intensity of the peak at −79.7 ppm can be explained by the lack of F^–^ inside of the solution since the hydrolysis of BF_4_^–^ is inhibited due to the low amount of H_2_O inside of the solution.

The presence of similar signals proves that, regardless of the used titanium precursor, similar detectable titanium complexes are built.

**Figure 10:**
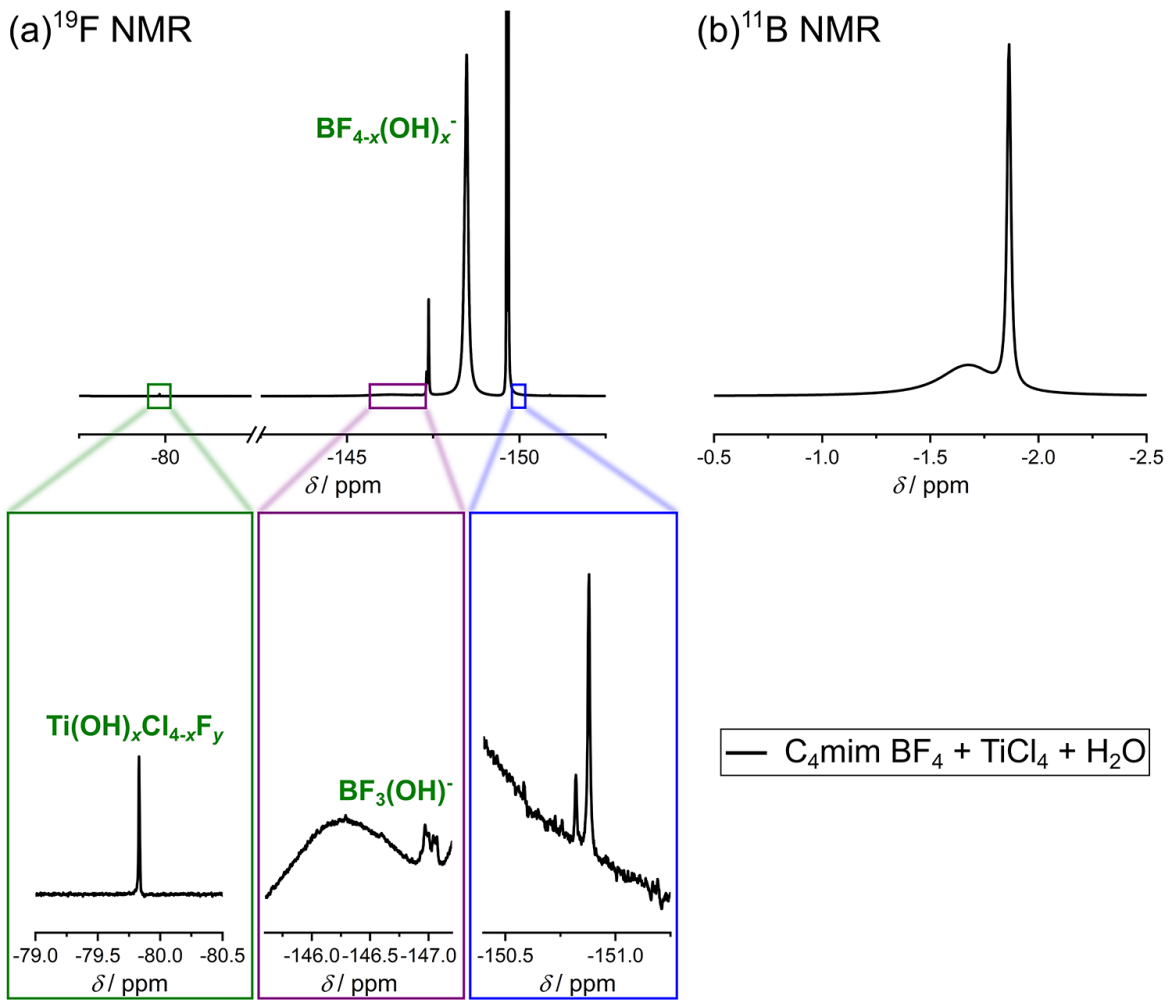
(a) ^19^F NMR and (b) ^11^B NMR spectrum of a mixture containing C_4_mim BF_4_, TiCl_4_, and H_2_O in a ratio of approximately 1:0.5:6.5. The chemicals were mixed at room temperature, and all spectra were measured with 400 MHz at 298 K, and a solution containing 0.1 M TFA in DMSO-*d*_6_ was used as external standard. For ^11^B NMR measurements, boron trifluoride etherate was used as reference.

After the addition of water in excess, the peak at −131.2 ppm can no longer be detected, while the signal at −79.7 ppm is still present (see Figure 9b). This observation was already noticed after the addition of water to a solution containing C_4_mim F, MeOH, and TiCl_4_ (see Figure 8b) which is another evidence that comparable complexes are built in both solutions. In the region from −145 to −151 ppm, again several peaks can be observed, but there are also a few changes detectable, compared with the spectrum measured for the solution without water (see Figure 9a). This can be explained by the fact that the hydrolysis of BF_4_^–^ can take place to a larger extent due to the larger amount of water, and thus different signals of the hydrolysis products (BF_4–_*_x_*OH*_x_*^−^) can occur. The cation of the IL is not affected in this solution (for ^1^H NMR and ^13^C NMR spectra, see Figure S10a,b), the ^11^B NMR spectrum (see Figure S10c) is comparable to the spectrum measured for C_4_mim BF_4_ + TiCl_4_ + H_2_O (see Figure 10b).

### Interactions inside of the Reaction Solution

Building on the insight provided by the comparative experiments described above, we now aim at understanding the reactions and interactions in the actual reaction solution used to synthesize Ti(OH)OF·0.66H_2_O. Since the color of this solution, i.e., C_4_mim BF_4_ + TiCl_4_ + H_2_O, is different from a solution containing only C_4_mim BF_4_ + TiCl_4_ (see Scheme 1), it can be assumed that the previously formed complexes of Ti with chlorine and BF_4_^–^ as ligands (see Figure 5) convert into other Ti complexes due to reactions with water. To peer further into the molecular structures and reactions, ^1^H, ^13^C, ^19^F, and ^11^B NMR spectra were measured. The ^1^H and ^13^C NMR spectra (see Figure S11) confirm the IL cation being unaffected since the spectra are comparable to the spectra of pure C_4_mim BF_4_ (see Figure 1a,b). The ^19^F and ^11^B NMR spectra, on the other hand, are different from the previously measured spectra. The ^11^B NMR spectrum (see Figure 10b) shows a broad and sharp signal in the range of approximately −1.5 to −2.0 ppm. Based on their position we attribute these signals to different B–F bonds. As discussed earlier, the chemical shift of boron bonds to chlorine is in a different range, and therefore it can be excluded that such compounds are present in this solution. The ^19^F NMR spectrum reveals several different signals (see Figure 10a). The peaks at −149.67 and −149.61 ppm are in conformity with BF_4_^–^ because of the high intensity and the integral ratio (1:0.25). This finding is quite interesting, if not amazing, because one would assume the more or less complete hydrolysis of BF_4_^–^ in such an acidic mixture. Note that these signals did not appear in a solution containing C_4_mim BF_4_ and TiCl_4_ (Figure 4). The only explanation for the BF_4_^–^ related ^19^F NMR signals coming up after the addition of water again is that complexes of Ti with BF_4_^–^as ligand (see Figure 5) were formed and that BF_4_^–^ is released from this kind of complexes upon addition of water. In addition, gas formation is observed after the addition of water to the reaction solution. The generated gas was HCl, which is formed in a reaction of the TiCl_4_ complex with water. Thus, after the addition of water, OH^–^ is bound to Ti. It is not possible to confirm if at this point of the reaction every Cl^–^ is replaced with OH^–^, therefore we refer to the resulting complex as Ti(OH)_*x*_Cl_4–*x*_F_*y*_.

The experimental observations are in good agreement with already published theoretical works, based on domain and Voronoi analyses.^24^ In these analyses, the molecules or ions, as well as possible functional groups, are divided into subsets. Thereby, the subsets are investigated with respect to their connectivity and their neighborhood behavior. In the already published study,^24^ the subsets were divided into polar, nonpolar, TiCl_4_ and water domains. It was found that the presence of water disturbs the microheterogeneous structure of the whole system, especially the microheterogeneous structure of the nonpolar and TiCl_4_ domains, which is manifested by a more scattered ordering of these domains. The addition of Voronoi analysis shows the surface coverage of the respective molecular or ionic moieties. In particular, for the system with all components, it is shown that the reference surfaces of titanium tetrachloride and tetrafluoroborate are largely covered by water and possible surface coverage by the cation is prevented. This disturbs the molecular order of the ionic liquid and possible interactions between cations and anions, and therefore the complexation between TiCl_4_ and BF_4_^–^ is prevented in the presence of water.

The ^19^F NMR peaks at about −147.0 ppm (see Figure 10a) can be assigned, based on the characteristic splitting of the peak (for reference, see Figure 3a), to the compound BF_3_(OH)^−^. The appearance of peaks, which can be assigned to different hydrolysis products of BF_4_^–^ (BF_4–*x*_OH_*x*_^–^), clearly proves that the hydrolysis must proceed via a different mechanism in the presence of TiCl_4_, since in this case the hydrolysis can take place quite fast at room temperature. It was not possible to detect hydrolysis products in a solution containing C_4_mim BF_4_ and H_2_O after a comparable waiting time (see Figure 3a), therefore it can be assumed that the hydrolysis proceeds only to a small extent via a direct interaction of BF_4_^–^ and H_2_O, instead it mainly occurs via interactions between BF_4_^–^ and OH^–^ ligands bound to titanium. This finding is in agreement with thermodynamical calculations already published in a previous study of our working group.^9^Table 2 summarizes the calculated interactions between BF_4–*x*_OH_*x*_^–^ and Ti(OH)_*y*_^*z*–^ out of the mentioned study.

**Table 2:** Thermodynamic Calculations of the Reaction Energy Δ*E* and the Free Reaction Enthalpy Δ*G* (in kJ/mol) of Interactions between BF_4–*x*_OH_*x*_^–^ and Ti(OH)_*y*_^*z*–^, Performed at a Temperature of 370 K, Taken from the Literature^9^^,^^a^

		Δ*E*	Δ*G*
(1)	[Ti(OH)_4_] + BF_4_^–^ → [Ti(OH)_3_F] + BF_3_(OH)^−^	–4.1	–2.0
(2)	[Ti(OH)_5_]^−^ + BF_4_^–^ → [Ti(OH)_4_F]^−^ + BF_3_(OH)^−^	–32.2	–29.7
(3)	[Ti(OH)_6_]^2–^ + BF_4_^–^ → [Ti(OH)_5_F]^2–^ + BF_3_(OH)^−^	–26.7	–28.9
(4)	[Ti(OH)_4_] + BF_3_(OH)^−^ → [Ti(OH)_3_F] + BF_2_(OH)_2_^–^	–2.5	–0.6
(5)	[Ti(OH)_5_]^−^ + BF_3_(OH)^−^ → [Ti(OH)_4_F]^−^ + BF_2_(OH)_2_^–^	–30.7	–28.3
(6)	[Ti(OH)_6_]^2–^ + BF_3_(OH)^−^ →[Ti(OH)_5_F]^2–^ + BF_2_(OH)_2_^–^	–25.1	–27.5
(7)	BF_4_^–^ + 2 H_2_O → BF_3_(OH)^−^ + F^–^ + H_3_O^+^	124.4	150.9

a Adapted with permission from Voepel, P.; et al., *Cryst. Growth Des*. **2017**, *17*, 5586–5601. Copyright 2017 American Chemical Society.

These calculations show that, from a thermodynamic point of view, the hydrolysis of BF_4_^–^ can proceed in this way since the values for Δ*E* and Δ*G* are negative for all of the calculated reactions. It should be noted that the calculations were performed for a temperature of 370 K, compared to the here applied temperature (298 K). However, we believe that the sign and magnitude of the values in Table 2 are not severely different to *T* = 298 K, as the entropic contribution Δ*S* is moderate. Also, the peak at −79.8 ppm observed in the ^19^F NMR spectrum (Figure 10a), which we relate to Ti–F bonds, supports the view that peaks at this position can be assigned to a titanium complex with OH^–^ and F^–^ as ligand. Hence, the theoretical thermodynamic parameters (see Table 2) and NMR data suggest that the hydrolysis of BF_4_^–^ does not occur just by the action of water, but requires the presence of Ti compounds in the solution. Interestingly the values of Δ*E* and Δ*G* for hydrolysis of BF_4_^–^ with water without TiCl_4_ at 370 K are positive (see Table 2(7)). Therefore, from a thermodynamic point of view, the hydrolysis of BF_4_^–^ with water is not favored. This explains the high amount of BF_4_^–^, which is still present after heating a solution with C_4_mim BF_4_ and H_2_O for 4 h at 95 °C (see Figure 3).

As the main outcome with respect to elucidating the overall synthesis, the hydrolysis of BF_4_^–^ can already take place at room temperature, mediated by the Ti compound, i.e., surprisingly the heating step is not crucial to spur the release of fluorine from BF_4_^–^. In addition, the presence of the peak at −79.8 ppm proves that Ti(OH)_*x*_Cl_4–*x*_F_*y*_ complexes are already present at room temperature, by systematically comparing the absence and presence of this signal in all measured solutions (see Table 3).

**Table 3:** Summary of the Solutions in Which the Signal at Approximately –79.8 ppm (^19^F NMR) Was Detected and in Which It Was Absent^a^

solution	peak at approx. –79.8 ppm? (^19^F NMR)	does the solution contain a titanium species?	does the solution contain H_2_O/MeOH?
C_4_mim BF_4_	no	no	no
C_4_mim BF_4_ + H_2_O	no	no	yes
C_4_mim BF_4_ + TiCl_4_	no	yes	no
C_4_mim BF_4_ + TiCl_4_ + H_2_O	yes (−79.83 ppm)	yes	yes
C_4_mim F + MeOH	no	no	yes
C_4_mim F + MeOH + TiCl_4_	yes (−79.85 ppm)	yes	yes
C_4_mim F + MeOH + TiCl_4_ + H_2_O	yes (−79.85 ppm)	yes	yes
C_4_mim BF_4_ + TTIP + HCl	yes (−79.67 ppm)	yes	yes
C_4_mim BF_4_ + TTIP + HCl + H_2_O	yes (−79.67 ppm)	yes	yes

a The presence/absence of this signal serves as proof for the presence of Ti(OH)_*x*_Cl_4–*x*_F_*y*_ in the reaction solution (C_4_mim BF_4_ + TiCl_4_ + H_2_O) already at room temperature.

It is noticeable that the peak at approx. −79.8 ppm can be detected in solutions containing an IL (C_4_mim BF_4_ or C_4_mim F), a titanium precursor (TiCl_4_ or TTIP), and H_2_O/MeOH, which is a clear proof that the peak can be assigned to Ti(OH)_*x*_Cl_4–*x*_F_*y*_ complexes (Ti(MeOH)*_x_*Cl_4–*x*_F_*y*_ if only MeOH is present).

**Figure 11:**
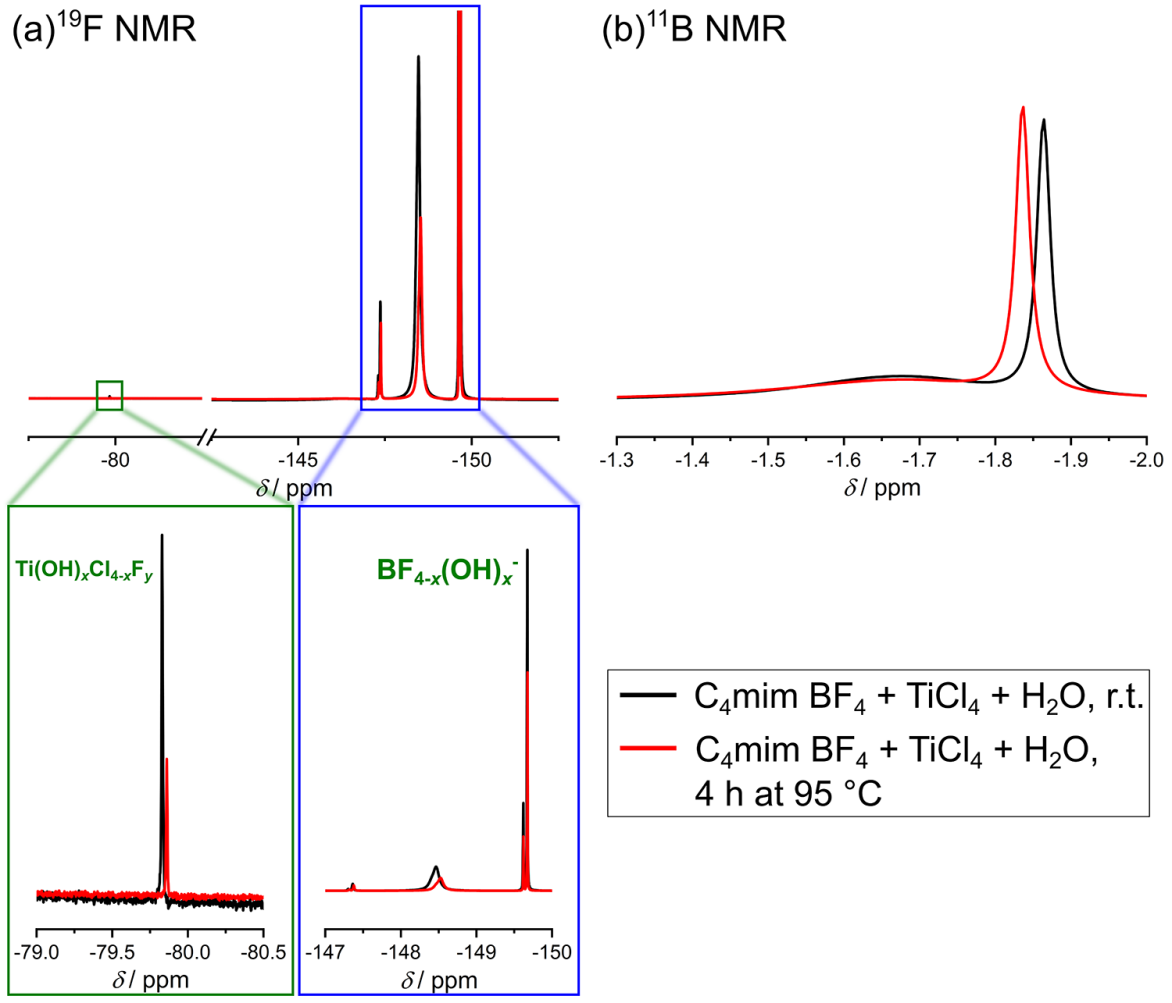
(a) ^19^F NMR and (b) ^11^B NMR spectrum of a mixture containing C_4_mim BF_4_, TiCl_4_, and H_2_O in a molar ratio of approximately 1:0.5:6.5. The solution was heated at 95 °C for 4 h, and after that, the solution was cooled down for the measurements. All spectra were measured with 400 MHz at 298 K, and a solution containing 0.1 M TFA in DMSO-*d*_6_ was used as external standard. For ^11^B NMR measurements, boron trifluoride etherate was used as reference.

We now focus on unraveling the heating step, which is inevitable to obtain Ti(OH)OF·0.66H_2_O. To tackle this question, the prepared reaction solution was heated up to 95 °C for 4 h (typical reaction time, see the Experimental Section) inside the NMR tube. After cooling down to room temperature, NMR spectra of the resulting solution were acquired. It should be noted that upon heating nanoparticles were formed, affecting the intensity of the NMR spectra. The ^1^H NMR and ^13^C NMR spectra (see Figure S12) prove that the IL cation is unaffected by the heating. Surprisingly, the ^19^F and ^11^B NMR spectra (see Figure 11) were comparable to the spectra measured prior to the heating step. As already discussed above, the heating step does therefore not substantially initiate or accelerate the hydrolysis of BF_4_^–^. Instead, the treatment at 95 °C induces the formation of Ti(OH)OF·0.66H_2_O nanoparticles out of the Ti(OH)_*x*_Cl_4–*x*_F_*y*_ complexes, i.e., the condensation of single Ti-containing entities into the crystalline array by the release of water. As already mentioned it is not possible to confirm if there are still Cl^–^ units present in the Ti complex prior to the heating step. However, X-ray photoelectron spectroscopy (XPS) and thermogravimetric analysis-mass spectrometry (TGA-MS) results of the finished product published in a previous study of our working group^11^ proved that no Cl^–^ is present within the product. Therefore, the remaining Cl^–^ must be released from the complex during the heating step.

**Figure 12:**
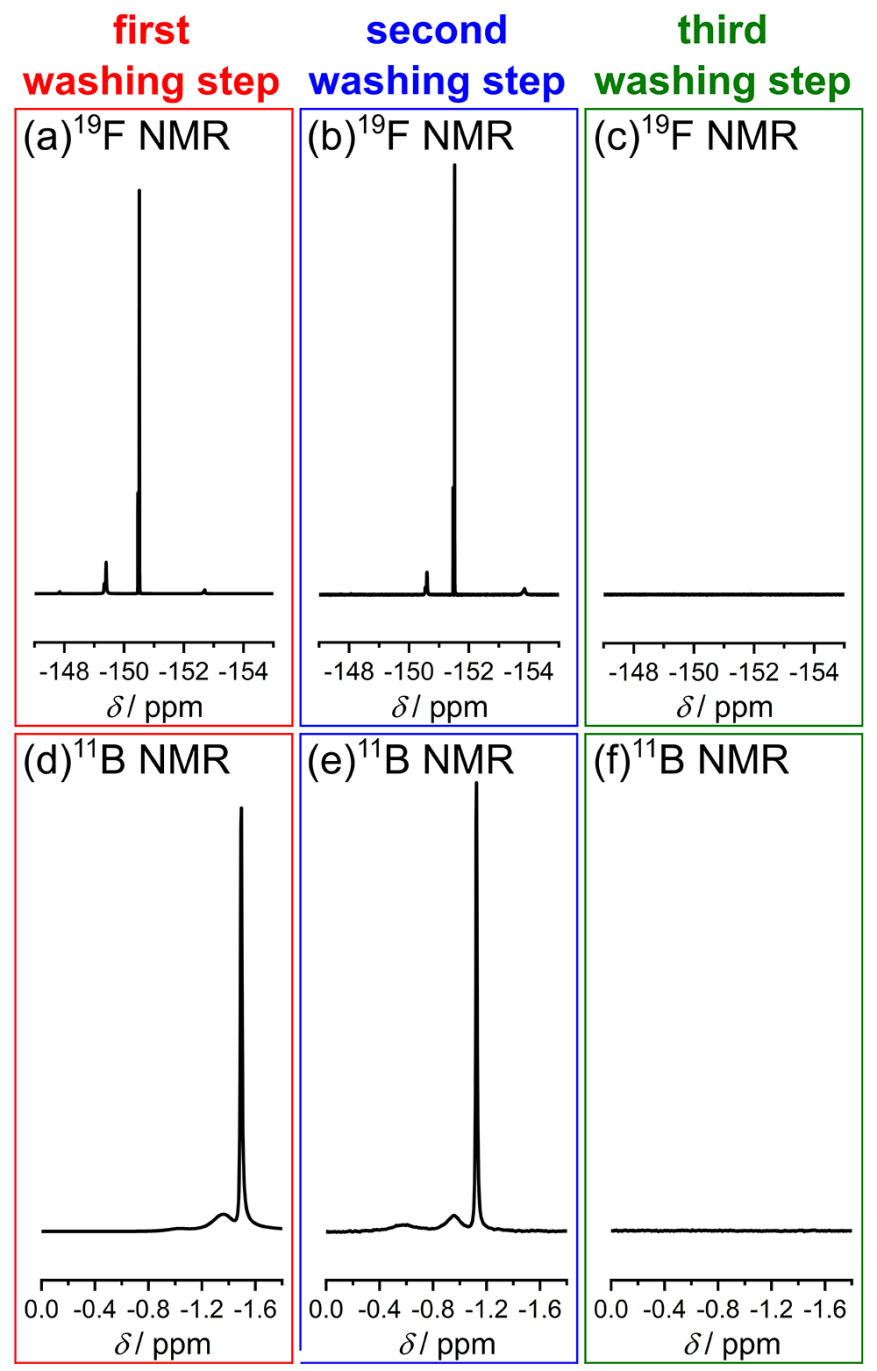
Washing steps of the produced nanoparticles. In each washing step, 2 mL of abs. EtOH was used. All spectra were measured with 400 MHz at 298 K, and a solution containing 0.1 M TFA in DMSO-*d*_6_ was used as external standard. For ^11^B NMR measurements, boron trifluoride etherate was used as reference.

Another approach to peer into the details of the synthesis is the analysis of the hydrolysis products after the reaction, and therefore, we analyzed the solutions obtained within the washing step as part of the synthesis (see the Experimental Section). Thus, we performed three washing steps after the synthesis and measured the NMR spectra of the washing solutions. Figure 12 shows relevant parts of the ^19^F and ^11^B NMR spectra, the full ^19^F NMR spectra and the ^1^H and ^13^C NMR spectra can be found in the SI file (see Figures S13–S15). In the first and second washing steps, hydrolysis products of BF_4_^–^ were detected, namely, BF_4–*x*_(OH)_*x*_^–^. It was possible to observe the same signals in both washing steps, although in comparison the position of each signal is shifted. The shift can be explained by a different concentration of the respective species. In the third washing step, in contrast, these species were no longer detectable. Since the spectra of the first two washing steps are comparable to the measured spectra of the reaction (see Figure 11), we can conclude that the washing steps are crucial for the purification of the different products, but they do not influence the reaction itself.

## Summary and Conclusions

In this work, we investigated the mechanism of an IL-based synthesis of the special fluorine-containing solid Ti(OH)OF·0.66H_2_O possessing a peculiar hexagonal tungsten bronze (HTB)-type structure. The synthesis is puzzling with respect to the simplicity of the procedure generating such a distinct crystalline solid, involving simple “beaker chemistry”, just heating a solution of commonplace chemicals, namely, the simple ionic liquid C_4_mim BF_4_, TiCl_4_, and H_2_O, as well as applying quite moderate temperature in the final heating step (95 °C). While previous studies had already indicated that the BF_4_^–^ anion plays a vital role by releasing fluorine and thus generating F-containing Ti clusters, the first reaction steps and products in this solution were still a matter of discussion. In particular, it had remained unclear which reaction steps take place in the mixture already at room temperature and which reactions are spurred by heating at 95 °C.

Here, we peered into the birth of the first Ti complexes generated upon reaction with BF_4_^–^ and H_2_O upon mixing already at room temperature, by the help of ^1^H, ^13^C, ^11^B, and ^19^F NMR spectroscopy measurements. For this purpose, we performed various NMR measurements of solutions with different, systematically varied compositions of the reactants. Surprisingly, in the first step of the reaction a complex containing TiCl_4_ and BF_4_^–^ is formed, already upon mixing at room temperature. Advanced quantum chemical calculations showed that, for instance, a binary TiCl_4_ complex, formed via side-linking by tetrafluoroborate and a TiCl_4_ complex with one coordinated BF_4_^–^ unit are plausible and possible. ^19^F NMR measurements performed on systematically varied solutions support these theoretical results in that the BF_4_^–^ ligand in such a complex is bound to titanium by a bridging fluorine atom: the bridging fluorine atom has a different shift range compared to the nonbridging fluorine atoms. This complexation is probably a crucial step for the synthesis, for instance, because it prevents the strong hydrolytic reaction between TiCl_4_ and H_2_O, which needs to be addressed by further theoretical studies.

After the addition of H_2_O to the solution containing IL and TiCl_4_, the signal originating from isolated BF_4_^–^ units appeared again in the ^19^F NMR spectrum, which shows that the complex between TiCl_4_ and BF_4_^–^ was destroyed, by forming Ti–O–bonds. In addition, a new peak at approximately −79.8 ppm (^19^F NMR spectrum) was detected, being attributable to a Ti(OH)_*x*_Cl_4–*x*_F_*y*_ complex. Again, the systematic comparison of ^19^F NMR spectra of different solutions provided ample evidence for this complex, as this peak is only detectable in solutions containing an IL, a titanium precursor, and H_2_O/MeOH (see Table 3). It is important to note that BF_4_^–^ does not undergo significant hydrolysis in H_2_O at room temperature. This finding is supported by positive Δ*G* values calculated for the reaction of BF_4_^–^ with water without TiCl_4_ at 370 K (see Table 2). Hence, it is the presence of the Ti species that initiates the decomposition of the BF_4_^–^ anion already at room temperature.

The experimental proof for such single Ti(OH)_*x*_Cl_4–*x*_F_*y*_ species being formed already at room temperature represents one of the major insights and advancements of this study, especially because it is difficult to predict the position of such signals even by advanced DFT methods. A further surprising result is thus the unexpected pronounced hydrolysis of BF_4_^–^ in the presence of Ti compounds, already at room temperature, while a mixture of C_4_mim BF_4_ and H_2_O exhibits a comparably slow formation of fluoride ions. This faster hydrolysis at room temperature is thus due to the fact that it occurs through interactions between Ti(OH)_*x*_ and BF_4_^–^ instead of interactions between H_2_O and BF_4_^–^. Since the hydrolysis of BF_4_^–^ therefore does not require the reaction temperature (95 °C), we conclude that the elevated temperature of 95 °C is only crucial for the condensation of the built Ti(OH)_*x*_Cl_4–*x*_F_*y*_ complexes, to overcome the activation energy and thus to spur the formation of Ti–O–Ti bonds and H_2_O.

**Scheme 2:**
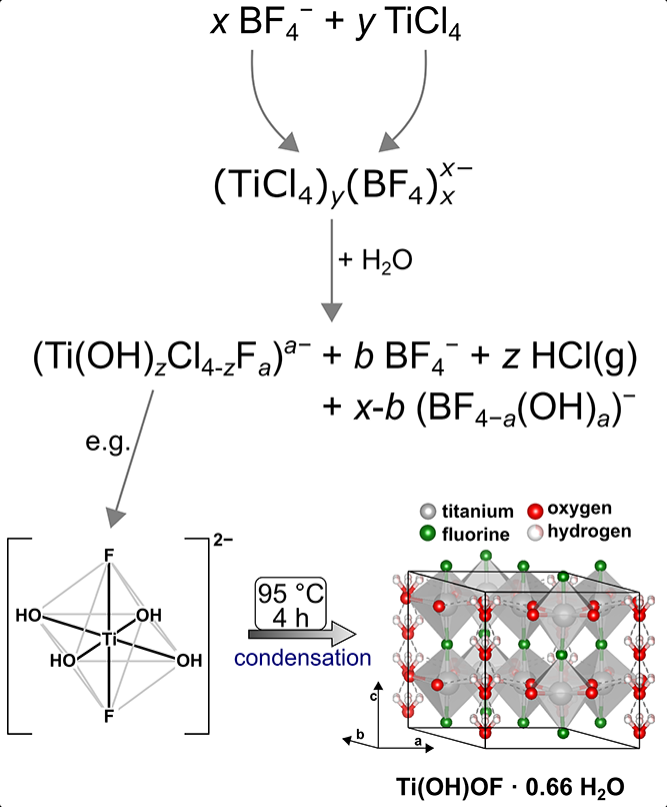
Schematic Illustration of the Investigated Reaction Mechanism

The crystal structure of Ti(OH)OF·0.66H_2_O has been investigated in detail in a previous work of our working group.^11^ With the help of XRD measurements and Rietveld refinements, it was proven that the crystal structure is built up of corner-sharing Ti(X)_6_ octahedra (X = O, F) and that the fluorine atoms occupy the apical positions of the built octahedra. To obtain this apical occupation in the product, it is therefore necessary that the fluorine atoms are arranged in trans position in the built Ti(OH)_*x*_Cl_4–*x*_F_*y*_ octahedra prior to the condensation step, as shown in Scheme 2. Based on these results, it is now possible to propose an adapted overall reaction mechanism for the investigated synthesis (see Scheme 2).

Hence, this study provides important insight into the first steps of the reaction, in particular, why the simple synthetic procedure is able to generate Ti(OH)OF·0.66H_2_O at moderate temperature, and to clarify the role of the IL: the IL provides fluorine in the form of the IL anion BF_4_^–^, spurs the hydrolysis of BF_4_^–^ already at room temperature in the presence of Ti species, and, moreover, is able to provide a stable solution of the involved compounds as well as the formed Ti complexes. In the light of previous works using imidazolium-based ILs for the synthesis of metal oxides, it is therefore justified to say that there is a specific “IL effect” for this type of synthesis, which involves the hydrolysis of transition-metal compounds. Hence, our study might help to develop strategies to synthesize other transition-metal oxyfluorides under ambient conditions.

## Experimental Section

### Synthesis Procedure

All ILs used in this work were purchased from IoLiTec (1-butyl-3-methylimidazolium tetrafluoroborate (C_4_mim BF_4_), purity: >99%, product code: IL-0012; 1-butyl-3-methylimidazolium fluoride methanol adduct (C_4_mim F), purity: 95% IL in methanol, product code CS-1608M). HCl, TiCl_4_, and titanium isopropoxide (TTIP, purity: 97%, product code: 205273) were purchased from Merck. All chemicals were used without further purification or modification.

In a typical synthesis, 3.85 mmol of IL (870.2 mg of C_4_mim BF_4_ or 609.2 mg of C_4_mim F) are heated up to 95 °C (80 °C for C_4_mim F) and mixed with 0.2 mL of TiCl_4_ (1.82 mmol). After stirring the yellow and transparent solution for at least 5 min, 0.45 mL (25 mmol) of H_2_O was added dropwise (caution: heavy reaction of TiCl_4_ with water under the release of HCl and potentially also HF). The solution was heated for 4 h at 95 °C (24 h at 80 °C for reactions with C_4_mim F). After a few minutes, it was possible to observe an increasing opacity of the reaction solution, indicating the formation of nanoparticles inside of the solution. The built nanoparticles were washed four times with technical ethanol and dried after the reaction suspension was cooled down. To prove that the received product is composed of Ti(OH)OF·0.66H_2_O and TiO_2_(B), we performed XRD measurements and Rietveld refinements of the synthesis product in the past.^12^ The results of these measurements can be found in the SI (see Figure S1 and Table S1). The single steps of this synthesis are documented by corresponding photographic images in the Supporting Information of ref (9).

In this work, we focus mainly on the interaction of different reactants. Therefore, solutions containing mixtures of different reactants, in the same ratio used in the described typical synthesis, were produced and NMR spectra were measured of these solutions (see Scheme 1).

### Synthesis with TTIP

In a typical synthesis, 870.2 mg of C_4_mim BF_4_ (3.85 mmol), 0.518 mL of TTIP (purity: 97%, 1.7 mmol), and 0.67 mL of conc. HCl_aq._ (37% solution, 21.87 mmol) were mixed at 95 °C. After stirring for at least 5 min, 0.45 mL of H_2_O (25 mmol) was added to the solution and the solution was heated for 4 h at 95 °C. After a few minutes, we observed an increasing opacity of the reaction solution, indicating the formation of nanoparticles inside of the solution. The nanoparticles were washed four times with technical ethanol and dried after the reaction suspension was cooled down.

### Preparation of NMR Samples

For the NMR measurements, a solution of 0.1 M trifluoroacetic acid (TFA) in DMSO-*d*_6_ was used as standard.^17^ To avoid interaction of the standard with the analyte, which could possibly distort the results, the analyte was sealed into a small capillary, which was then placed inside of an NMR tube. Figure S16 shows a schematic illustration of the prepared NMR samples. For some samples, it was not possible to use the 0.1 M TFA-in-DMSO-*d*_6_ solution as a standard since the peak intensity of the compounds was too low (e.g., Figure 8). In these cases, pure DMSO-*d*_6_ was used inside of the capillary and trichlorofluoromethane was used as a standard for the ^19^F NMR measurements.

### Instrumental Settings

The NMR spectra were measured with a Bruker Avance III 400 MHz HD and a Bruker Avance II 400 MHz spectrometer. All spectra were measured with 400 MHz at 298 K. The chemical shifts δ are reported in parts per million (ppm) relative to the solvent signal of DMSO-*d*_6_ (^1^H and ^13^C NMR measurements) or the solvent signal of the 0.1 M TFA in DMSO-*d*_6_ solution (^19^F NMR measurements, the shift of this external standard solution was reported in the literature).^17^ For ^11^B NMR measurements, boron trifluoride etherate was used as reference. The coupling constants *J* for ^1^H NMR measurements can be found in the SI.

### Computational Details: Static Quantum Chemical Calculations

The starting geometries of the structures to be investigated were initially built using the software package MOLDEN (version 5.4).^18^ Subsequently, all quantum chemical calculations were performed using the ORCA 4.0 program.^19^ The geometry-optimized structures were performed using the B3LYP functional^20−22^ and the def2-TZVPP basis set.^23^ Tight convergence criteria were applied for the SCF cycle and geometry optimization. To verify if the obtained structures were ground states, it was ensured that the Hessian did not have negative eigenvalues for minima. To take into account the solvation effects, approximations were performed with the conduction-like polarizable continuum model (CPCM), which is also possible with the ORCA program with the functional and basis sets mentioned above. A dielectric constant of 38.3 was used, which reflects well the mixture of water and C_4_mim-based ionic liquids.

The experimentally obtained results are compared not only with static DFT calculations but also with the results of theoretical investigations of so-called ab initio molecular dynamic (AIMD) simulations previously published.^24^ The more detailed description, explanation, and setup, as well as the procedure of these are explained in detail in this recent publication.^24^ The compositions of the simulation boxes reflect the compositions of the used reaction mixtures.

## Supporting Information Available

XRD spectra, results of Rietveld refinement, scheme with all measured solutions, additional NMR spectra, list of all peaks measured in different NMR spectra, coupling constants *J* for ^1^H NMR measurements, structural formula of two reference materials, results of quantum chemical calculations, and *xyz* coordinates of the optimized structures (PDF)

## Abbreviations

AIMD: ab initio molecular dynamic
C_4_mim BF_4_: 1-butyl-3-methylimidazolium tetrafluoroborate
C_4_mim F: 1-butyl-3-methylimidazolium fluoride
C_16_mim Cl: 1-hexadecyl-3-methylimidazolium chloride
CPCM: conductor-like polarizable continuum model
DEME BF_4_^–^: *N*,*N*-diethyl-*N*-methyl-*N*-(2-methoxyethyl)ammonium tetrafluoroborate)
DFT: density functional theory
DMSO-*d*_6_: dimethyl sulfoxide-*d*_6_
HTB: hexagonal tungsten bronze
IL: ionic liquid
NMR: nuclear magnetic resonance
ppm: parts per million
RDFs: radial distribution functions
TFA: trifluoroacetic acid
TGA-MS: thermogravimetric analysis-mass spectrometry
TTIP: titanium isopropoxide
XPS: X-ray photoelectron spectroscopy
XRD: X-ray diffraction
