# Supplementary material for: Ionic Liquid-Based Low-Temperature Synthesis of Crystalline Ti(OH)OF·0.66H2O: Elucidating the Molecular Reaction Steps by NMR Spectroscopy and Theoretical Studies
Source: ACS Omega. 2022 Feb 2;7(6):5350–65. doi: 10.1021/acsomega.1c06534 (PMC8851441; doi:10.1021/acsomega.1c06534)
Supplement: Supplementary file 1 — ao1c06534_si_001.pdf [file ao1c06534_si_001.pdf]

Supporting Information:

Ionic Liquid-based Low-temperature Synthesis of Crystalline $Ti(OH)OF \cdot 0.66\ H_2O$: Elucidating the Molecular Reaction Steps by NMR Spectroscopy and Theoretical Studies

*Melanie Sieland[1], Manuel Schenker[1], Lars Esser[2], Barbara Kirchner[2], Bernd M. Smarsly[1,3]**

[1]Institute of Physical Chemistry, Justus Liebig University, Heinrich-Buff-Ring 17, D-35392 Giessen, Germany

[2] Mulliken Center for Theoretical Chemistry, University of Bonn, Beringstrasse 4+6, D-53115 Bonn, Germany

[3]Center of Materials Research, Justus Liebig University, Heinrich-Buff-Ring 16, D-35392 Giessen, Germany

NMR spectroscopy

Multiplicities are written as: s = singlet, d = doublet, t = triplet, q = quartet, quint = quintet, sext = sextet, m = multiplet

All spectra were measured at 298 K.

1-Butyl-3-methylimidazolium tetrafluoroborat (C_4mim BF_4)

^{1}H NMR (400 MHz, external reference: 0.1 M Trifluoroacetic acid (TFA) in DMSO-d_6)**:** δ = 7.98 (s, 1H), 6.90 (m, 1H), 6.84 (m, 1H), 3.53 (t, *J* = 7.2 Hz , 2H), 3.25 (s, 3H), 1.14 (quint, *J* = 7.4 Hz, 2H), 0.58 (sext, *J* = 7.3 Hz, 2H), 0.15 (t, *J* = 7.4 Hz , 3H)

^{13}C NMR (400 MHz, external reference: 0.1 M Trifluoroacetic acid (TFA) in DMSO-d_6)**:** δ = 135.45, 122.32, 121.00, 47.94, 34.34, 30.45, 17.80, 11.58

^{19}F NMR (400 MHz, external reference: 0.1 M Trifluoroacetic acid (TFA) in DMSO-d_6)**:**

δ = -74.95 (TFA), -150.16, -150.21

^{11}B NMR (400 MHz, external reference: 0.1 M Trifluoroacetic acid (TFA) in DMSO-d_6):

δ = -1.62

C_4mim BF_4 + H_2O

^{1}H NMR (400 MHz, external reference: 0.1 M Trifluoroacetic acid (TFA) in DMSO-d_6):

δ = 8.01 (s, 1H), 6.87 (m, 1H), 6.83 (m, 1H), 3.72 (s, H_2O), 3.56 (t, *J* = 7.3 Hz , 2H), 3.28 (s, 3H), 1.19 (m, 2H), 0.66 (sext, *J* = 7.3 Hz, 2H), 0.23 (t, *J* = 7.4 Hz , 3H)

^{13}C NMR (400 MHz, external reference: DMSO-d_6): δ = 134.93, 122.45, 121.12, 48.15, 34.48, 30.38, 17.79, 11.03

^{19}F NMR (400 MHz, external reference: 0.1 M Trifluoroacetic acid (TFA) in DMSO-d_6):

δ = -74.95 (TFA), -150.21, -150.27

^{11}B NMR (400 MHz, external reference: 0.1 M Trifluoroacetic acid (TFA) in DMSO-d_6):

δ = -1.80

C_4mim BF_4 + H_2O, heated for 4 h at 95 °C

^{1}H NMR (400 MHz, external reference: 0.1 M Trifluoroacetic acid (TFA) in DMSO-d_6):

δ = 8.01 (m, 1H), 6.87 (m, 1H), 6.83 (m, 1H), 3.55 (m, 2H, H_2O), 3.27 (s, 3H), 1.17 (m, 2H), 0.63 (m, 2H), 0.21 (t, *J* = 7.4 Hz , 3H)

^{13}C NMR (400 MHz, external reference: DMSO-d_6): δ = 134.95, 122.45, 121.11, 48.13, 34.48, 30.38, 17.79, 11.60

^{19}F NMR (400 MHz, external reference: 0.1 M Trifluoroacetic acid (TFA) in DMSO-d_6):

δ = -74.95 (TFA), -128.00, -143.66 (m), -150.25

^{11}B NMR (400 MHz, external reference: 0.1 M Trifluoroacetic acid (TFA) in DMSO-d_6):

δ = -0.26 (q), -1.77

C_4mim BF_4 + $TiCl_4$

^{1}H NMR (400 MHz, external reference: 0.1 M Trifluoroacetic acid (TFA) in DMSO-d_6):

δ = 8.16 (s, 1H), 7.06 (m, 1H), 7.01 (m, 1H), 3.78 (m, 2H), 3.52 (s, 3H), 1.42 (m, 2H), 0.87 (m, 2H), 0.42 (t, *J* = 7.2 Hz, 3H)

^{13}C NMR (400 MHz, external reference: 0.1 M Trifluoroacetic acid (TFA) in DMSO-d_6):

δ = 135.09, 122.78, 121.52, 48.72, 35.13, 30.95, 18.36, 12.18

^{19}F NMR (400 MHz, external reference: 0.1 M Trifluoroacetic acid (TFA) in DMSO-d_6):

δ = -74.95 (TFA), -131.34, 135.48 to -150.12

^{11}B NMR (400 MHz, external reference: 0.1 M Trifluoroacetic acid (TFA) in DMSO-d_6)**:**
δ = -1.03

C_4mim F + MeOH
^{1}H NMR (400 MHz, external reference: 0.1 M Trifluoroacetic acid (TFA) in DMSO-d_6)**:**
δ = 8.16 (m, 1H), 7.99 (m, 1H), 6.28 (MeOH), 3.87 (t, *J* = 7.2 Hz, 2H), 3.67 (m), 3.60 (s, 3H), 2.65 (MeOH), 1.31 (quint, *J* = 7.2 Hz, 2H), 0.68 (sext, *J* = 7.2 Hz, 2H), 0.26 (t, *J* = 7.3 Hz, 3H)
^{13}C NMR (400 MHz, external reference: 0.1 M Trifluoroacetic acid (TFA) in DMSO-d_6)**:**
δ = 138.82, 123.34, 122.38, 47.63, 47.35, 34.24, 31.23, 18.19, 11.96
^{19}F NMR (400 MHz, external reference: 0.1 M Trifluoroacetic acid (TFA) in DMSO-d_6)**:**
δ = -74.95 (TFA), -108.66, -145.33

C_4mim F + MeOH + $TiCl_4$
^{1}H NMR (400 MHz, external reference: DMSO-d_6)**:** δ = 8.85 (s, 1H), 8.05 (MeOH), 7.40 (s, 1H), 7.31 (s, 1H), 3.85 (m, 2H), 3.57 (s, 3H), 3.06 (MeOH), 1.33 (m ,2H), 0.74 (m, 2H), 0.29 (t, *J* = 7.3 Hz , 3H)
^{13}C NMR (400 MHz, external reference: DMSO-d_6)**:** δ = 135.88, 122.79, 122.62, 48.31, 35.39, 31.18, 26.18, 25.68, 18.28, 12.34
^{19}F NMR (400 MHz, external reference: DMSO-d_6)**:**
δ = -79.85, -131.28, -150.30

C_4mim F + MeOH + $TiCl_4$ + H_2O
^{1}H NMR (400 MHz, external reference: DMSO-d_6)**:** δ = 8.31 (s, 1H), 7.04 (m, 1H), 6.99 (m, 1H), 5.92 (H_2O), 3.68 (t, *J* = 7.2 Hz, 2H), 3.40 (s, 3H), 2.86 (MeOH), 1.27 (m , 2H), 0.72 (m, 2H), 0.31 (t, *J* = 7.4 Hz , 3H)
^{13}C NMR (400 MHz, external reference: DMSO-d_6)**:** δ = 134.84, 122.77, 121.49, 48.49, 35.31, 30.61, 18.07, 12.19
^{19}F NMR (400 MHz, external reference: DMSO-d_6)**:**
δ = -79.85, -150.18

C_4mim BF_4 + TTIP + conc. HCl

^{1}H NMR (400 MHz, external reference: 0.1 M Trifluoroacetic acid (TFA) in DMSO-d_6):

δ = 8.22 (s, 1H), 7.03 to 6.96 (m, 2H + H_2O), 3.69 (t, J = 7.2 Hz , 2H), 3.42 (s, 3H), 1.31 (m, 2H), 0.78 (m, 2H), 0.69 (d, J = 6.2 Hz, R-iOPr), 0.35 (t, J = 7.4 Hz , 3H)

^{13}C NMR (400 MHz, external reference: 0.1 M Trifluoroacetic acid (TFA) in DMSO-d_6):

δ = 134.91, 122.78, 121.44, 48.50, 35.16, 30.73, 22.78, 18.10, 12.11

^{19}F NMR (400 MHz, external reference: 0.1 M Trifluoroacetic acid (TFA) in DMSO-d_6):

δ = -74.95 (TFA), 79.67, -131.21, -147.09, -147.14, -148.31, -149.46, -149.52, -150.02, -150.08

^{11}B NMR (400 MHz, external reference: 0.1 M Trifluoroacetic acid (TFA) in DMSO-d_6):

δ = -1.59, -1.72

C_4mim BF_4 + TTIP + conc. HCl + H_2O

^{1}H NMR (400 MHz, external reference: 0.1 M Trifluoroacetic acid (TFA) in DMSO-d_6):

δ = 8.20 (s, 1H), 6.99 (m, 1H), 6.96 (m, 1H), 5.86 (H_2O), 3.67 (t, J = 7.2 Hz , 2H), 3.54 (m, R-iOPr), 3.40 (s, 3H), 1.29 (m, 2H), 0.76 (m, 2H), 0.64 (d, J = 6.1 Hz, R-iOPr), 0.35 (t, J = 7.4 Hz , 3H)

^{13}C NMR (400 MHz, external reference: 0.1 M Trifluoroacetic acid (TFA) in DMSO-d_6):

δ = 134.82, 122.77, 121.41, 64.34, 48.46, 35.06, 30.63, 23.02, 18.04, 12.04

^{19}F NMR (400 MHz, external reference: 0.1 M Trifluoroacetic acid (TFA) in DMSO-d_6):

δ = -74.95 (TFA), -79.67, -147.24, -147.29, -148.08, -148.14, -149.72, -149.77

^{11}B NMR (400 MHz, external reference: 0.1 M Trifluoroacetic acid (TFA) in DMSO-d_6):

δ = -1.55, -1.78

C_4mim BF_4 + $TiCl_4$ + H_2O

^{1}H NMR (400 MHz, external reference: 0.1 M Trifluoroacetic acid (TFA) in DMSO-d_6):

δ = 8.00 (s, 1H), 6.90 to 6.82 (H_2O + 2H), 3.56 (t, J = 7.2 Hz , 2H), 3.29 (s, 3H), 1.18 (quint, J = 7.3 Hz, 2H), 0.63 (sext, J = 7.4 Hz, 2H), 0.21 (t, J = 7.3 Hz , 3H)

^{13}C NMR (400 MHz, external reference: 0.1 M Trifluoroacetic acid (TFA) in DMSO-d_6):

δ = 134.64, 122.59, 121.24, 48.35, 35.00, 30.45, 17.89, 11.87

^{19}F NMR (400 MHz, external reference: 0.1 M Trifluoroacetic acid (TFA) in DMSO-d_6):

δ = -74.95 (TFA), -79.83, -146.26, -147.02, -147.30, -147.36, -148.46, -149.61, -149.67, -150.82, -150.88

^{11}B NMR (400 MHz, external reference: 0.1 M Trifluoroacetic acid (TFA) in DMSO-d_6):

δ = -1.67, -1.86

C_4mim BF_4 + $TiCl_4$ + H_2O, heated for 4 h at 95 °C

^{1}H NMR (400 MHz, external reference: 0.1 M Trifluoroacetic acid (TFA) in DMSO-d_6): δ = 8.19 (s, 1H), 7.05 to 7.00 (m, 2H), 6.86 (H_2O) 3.73 (t, *J* = 7.2 Hz , 2H), 3.47 (s, 3H), 1.35 (m, 2H), 0.80 (m, 2H), 0.38 (t, *J* = 7.4 Hz , 3H)

^{13}C NMR (400 MHz, external reference: 0.1 M Trifluoroacetic acid (TFA) in DMSO-d_6): δ = 134.70, 122.58, 121.23, 48.32, 34.97, 30.46, 17.89, 11.84

^{19}F NMR (400 MHz, external reference: 0.1 M Trifluoroacetic acid (TFA) in DMSO-d_6): δ = -74.95 (TFA), -79.86, -147.31, -147.37, -148.46, -148.52 -149.62, -149.67

^{11}B NMR (400 MHz, external reference: 0.1 M Trifluoroacetic acid (TFA) in DMSO-d_6): δ = -1.67, -1.84

Washing step 1

^{1}H NMR (400 MHz, external reference: 0.1 M Trifluoroacetic acid (TFA) in DMSO-d_6): δ = 8.53 (s, 1H), 7.25 (m, 1H), 7.19 (m, 1H), 6.41 (H_2O), 5.64 (EtOH), 3.86 (t, *J* = 7.2 Hz , 2H), 3.57 (s, 3H), 3.26 (q, 7.0 Hz, 2H, EtOH), 1.47 (quint, *J* = 7.4 Hz, 2H), 0.95 (sext, *J* = 7.3 Hz, 2H), 0.78 (t, *J* = 7.0 Hz, 3H, EtOH), 0.53 (t, *J* = 7.3 Hz , 3H)

^{13}C NMR (400 MHz, external reference: 0.1 M Trifluoroacetic acid (TFA) in DMSO-d_6): δ = 135.40, 122.83, 121.56, 56.70, 48.50, 34.97, 30.95, 18.23, 16.40, 12.05

^{19}F NMR (400 MHz, external reference: 0.1 M Trifluoroacetic acid (TFA) in DMSO-d_6): δ = -74.95 (TFA), -79.98, -147.78, -147.84, -149.33, -149.39, -150.46, -150.51, -152.63, -152.69

^{11}B NMR (400 MHz, external reference: 0.1 M Trifluoroacetic acid (TFA) in DMSO-d_6): δ = -1.01, -1.36, -1.49

Washing step 2

^{1}H NMR (400 MHz, external reference: 0.1 M Trifluoroacetic acid (TFA) in DMSO-d_6): δ = 9.06 (s, 1H), 7.69 (m, 1H), 7.62 (m, 1H), 5.43 (EtOH), 4.24 (t, *J* = 7.3 Hz , 2H), 3.95 (s, 3H), 3.59 (q, 7.0 Hz, 2H, EtOH), 1.86 (m, 2H), 1.36 (m, 2H), 1.15 (t, *J* = 7.0 Hz, 3H, EtOH), 0.95 (t, *J* = 7.4 Hz , 3H)

^{13}C NMR (400 MHz, external reference: 0.1 M Trifluoroacetic acid (TFA) in DMSO-d_6): δ = 136.02, 116.59, 113.72, 56.44, 56.05, 48.82, 35.12, 31.44, 18.56, 16.96, 12.27

^{19}F NMR (400 MHz, external reference: 0.1 M Trifluoroacetic acid (TFA) in DMSO-d_6):

δ = -74.95 (TFA), -150.54, -150.60, -151.47, -151.52, -153.85

^{11}B NMR (400 MHz, external reference: 0.1 M Trifluoroacetic acid (TFA) in DMSO-d_6)**:**

δ = -0.58, -0.95, -1.12

Washing step 3

^{1}H NMR (400 MHz, external reference: 0.1 M Trifluoroacetic acid (TFA) in DMSO-d_6)**:**

δ = 5.36 (s, 1H), 5.19, 3.63 (q, J = 7.0 Hz, 2H), 1.19 (t, J = 7.0 Hz, 3H)

^{13}C NMR (400 MHz, external reference: 0.1 M Trifluoroacetic acid (TFA) in DMSO-d_6)**:**

δ = 56.39, 16.99

^{19}F NMR (400 MHz, external reference: 0.1 M Trifluoroacetic acid (TFA) in DMSO-d_6)**:**

δ = -74.95 (TFA)

^{11}B NMR (400 MHz, external reference: 0.1 M Trifluoroacetic acid (TFA) in DMSO-d_6)**:**

δ = no peaks

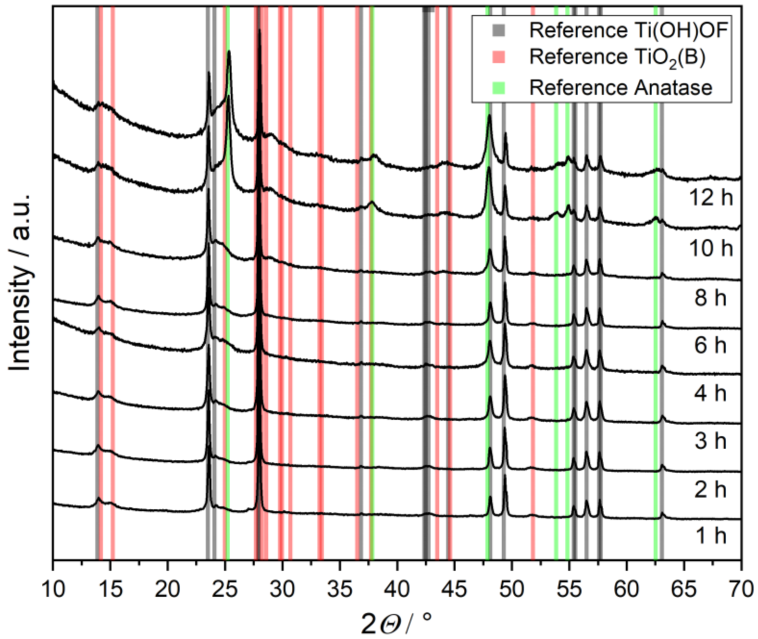

Figure S1. Time dependent XRD study of a synthesis leading to the formation of Ti(OH)OF · 0.66 H_2O. In this work the synthesis was stopped after 4 h synthesis time. Reprinted with permission from M. Sieland, V. Camus-Genot, I. Djerdj, B. M. Smarsly, *ChemistryOpen* **2021**, *10* (2), 181–188. (DOI: https:/doi.org/10.1002/open.202000256) Copyright © 2020 The Authors. Published by Wiley-VCH GmbH.

Table S1. Results of the Rietveld refinement performed for the synthesis which was used in the recent study as well.

Reprinted with permission from M. Sieland, V. Camus-Genot, I. Djerdj, B. M. Smarsly, *ChemistryOpen* **2021**, *10* (2), 181–188. (DOI: https:/doi.org/10.1002/open.202000256) Copyright © 2020 The Authors. Published by Wiley-VCH GmbH.

**ex-situ study with C_4mim BF_4, 4 h**	Ti(OH)OF · 0.66 H_2O	TiO_2(B)
Lattice parameters (Å)	a = 7.3936(4), c = 7.5810(4)	a = 12.661(5), b = 3.780(1), c = 6.102(3), β = 106.65(6)°
Cell volume (Å^3)	358.90(3)	279.8(2)
Calculated density (g cm^{-3})	3.078	3.792
No. of parameters refined	86	
No. of bond lengths restrained	20	18
No. of bond angles restrained	0	0
Average apparent crystallite size (nm)	27.5(9.3)	5.3
Average apparent microstrain (x 10^{-4})	Not extracted	Not extracted
Phase composition (wt%)	67.53(3.53)	32.47(4.65)
R_B (%)	6.57	4.09
Conventional R_p, R_{wp}, R_e (%)	17.1, 17.1, 6.42	
GoF	2.6	

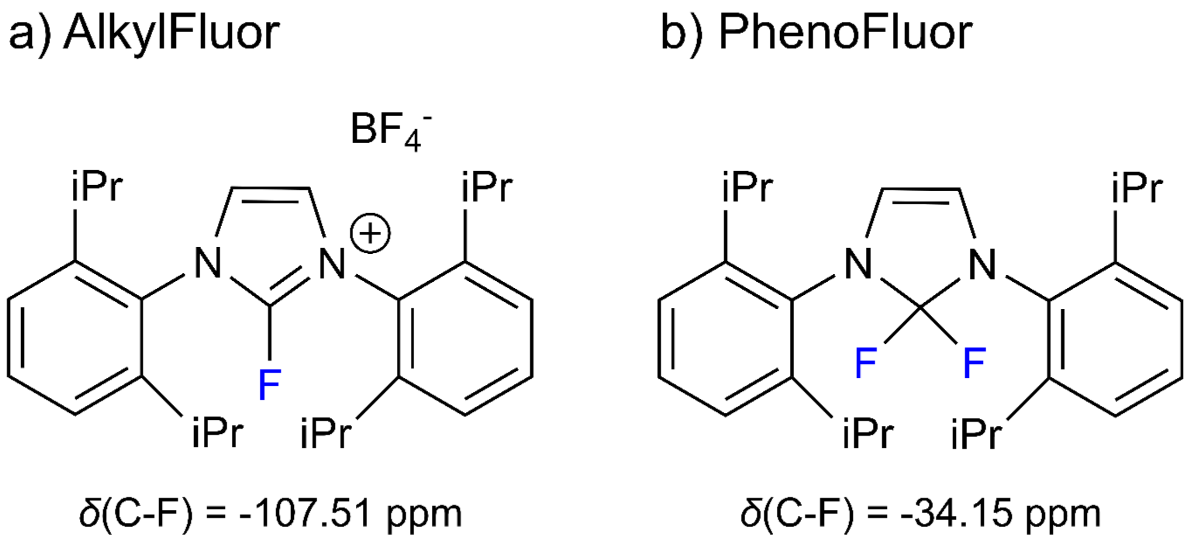

Figure S2. Structural formula of (a) AlkylFluor and (b) Phenofluor and the chemical shift of the C-F bonds for ^{19}F NMR measurements. The chemical shifts are based on C. P. Rosenau et al. *Angew. Chem. Int. Ed.* **2018**, 57, 9528-9533.

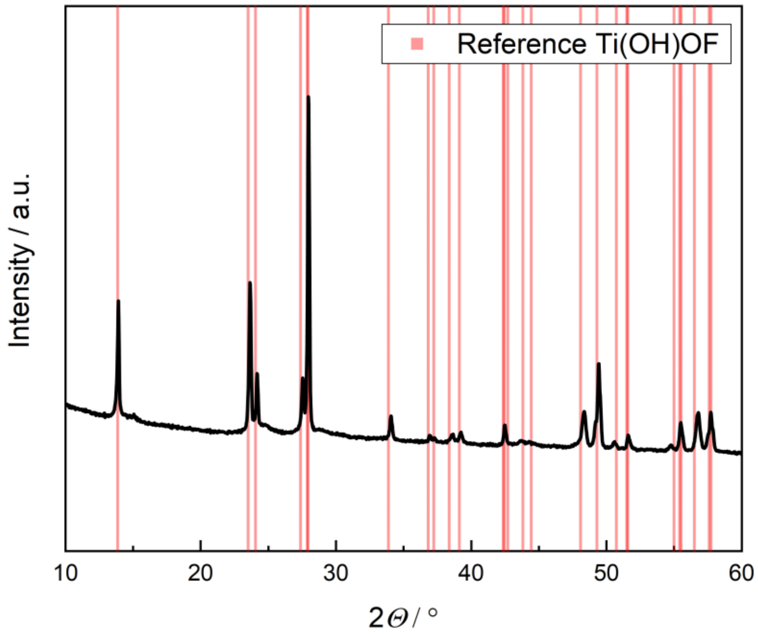

Figure S3. Powder XRD patterns and reference pattern (solid bars) of the product received after 4 h in a synthesis with TTIP. The reference pattern is based on B. Li et al., *Nanoscale Res. Lett.* **2015**, *10*, 1-7.

Scheme S1. All measured solutions and questions we tried to answer with different NMR measurements.

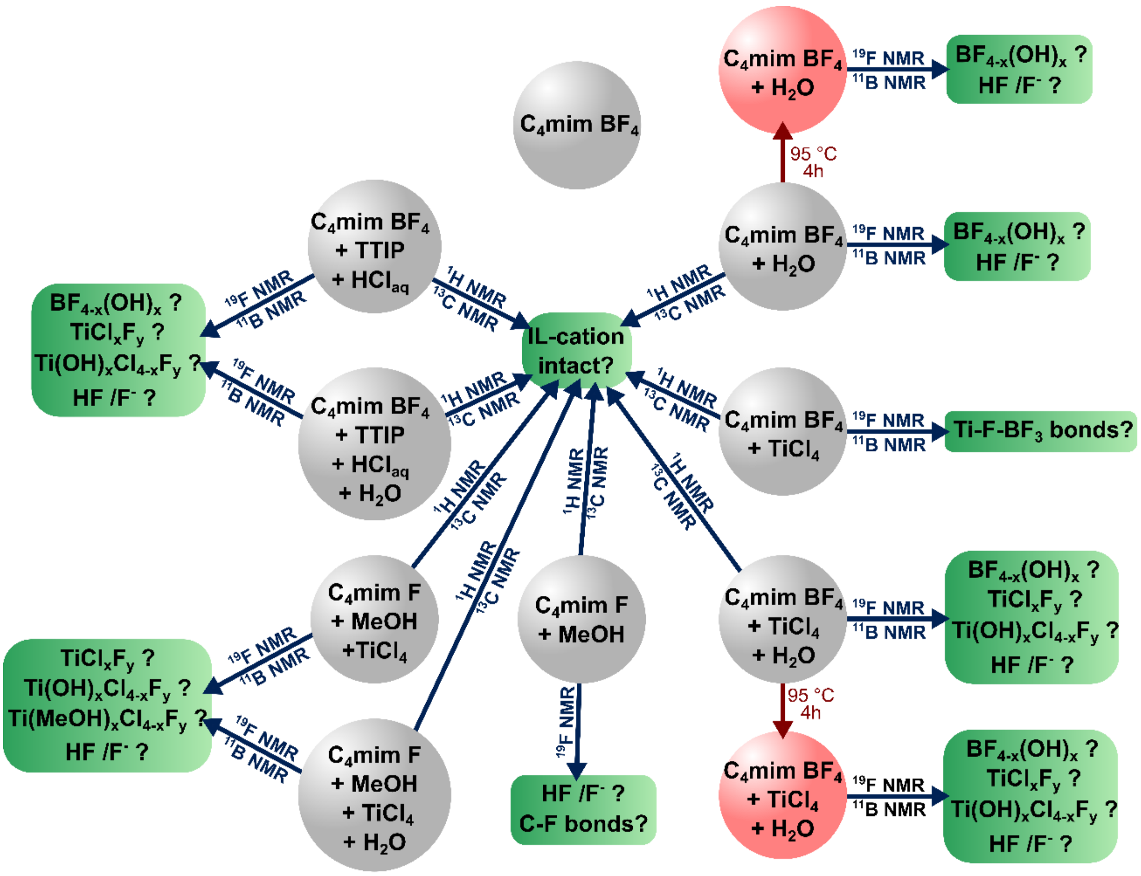

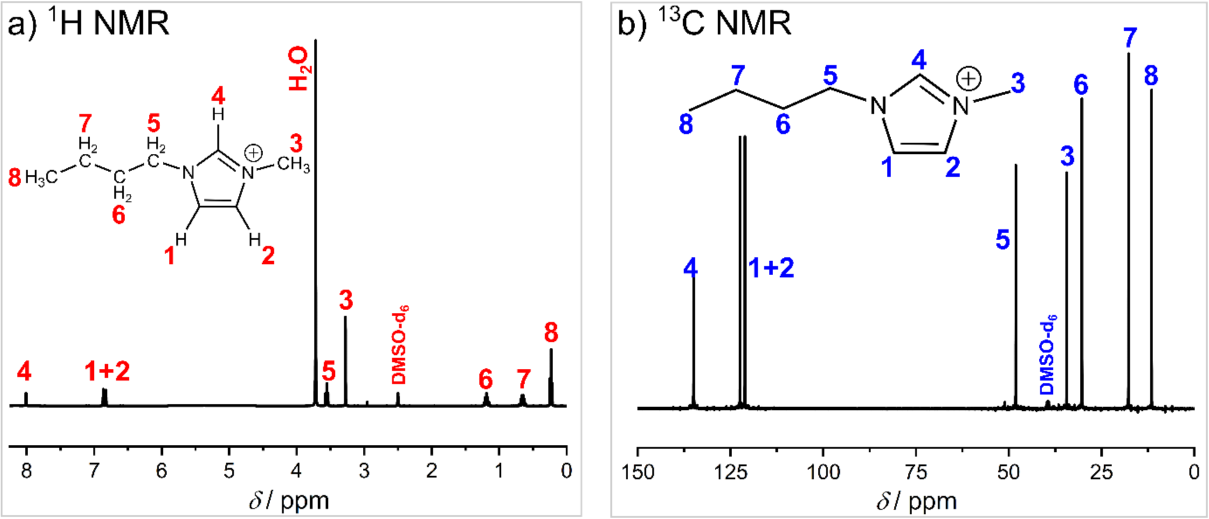

Figure S4. (a) ^{1}H NMR and (b) ^{13}C NMR spectrum of a mixture containing C_4mim BF_4 and H_2O with a molar ratio of 1:6.5. The chemicals were mixed at room temperature, all spectra were measured with 400 MHz at 298 K, and a solution containing 0.1 M TFA in DMSO-d_6 was used as external standard.

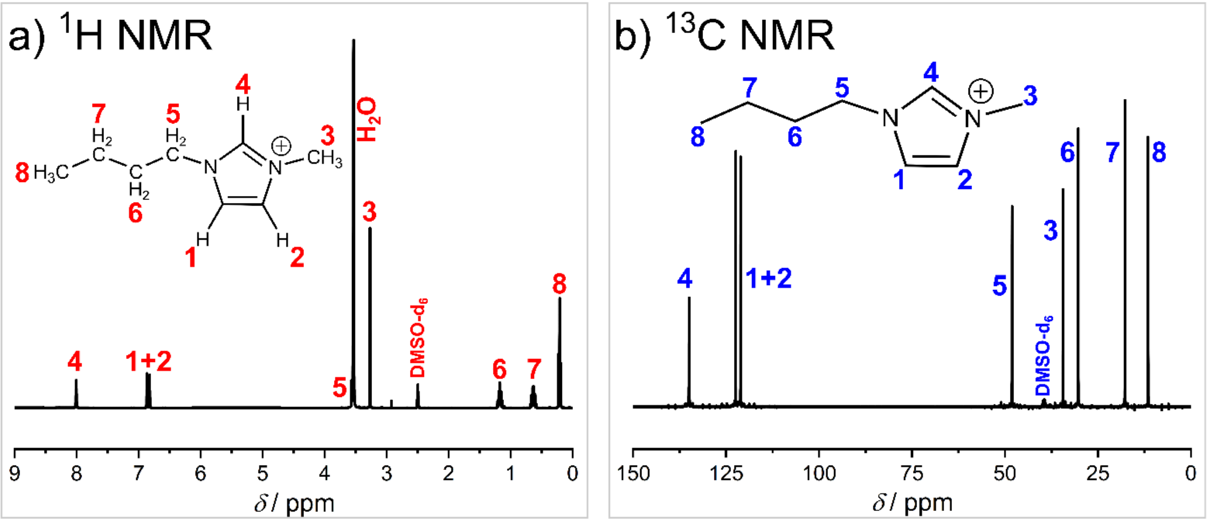

Figure S5. (a) ^{1}H NMR and (b) ^{13}C NMR spectrum of a mixture containing C_4mim BF_4 and H_2O with a molar ratio of 1:6.5 which was heated to 95 °C for 4 h. All spectra were measured with 400 MHz at 298 K, and a solution containing 0.1 M TFA in DMSO-d_6 was used as external standard.

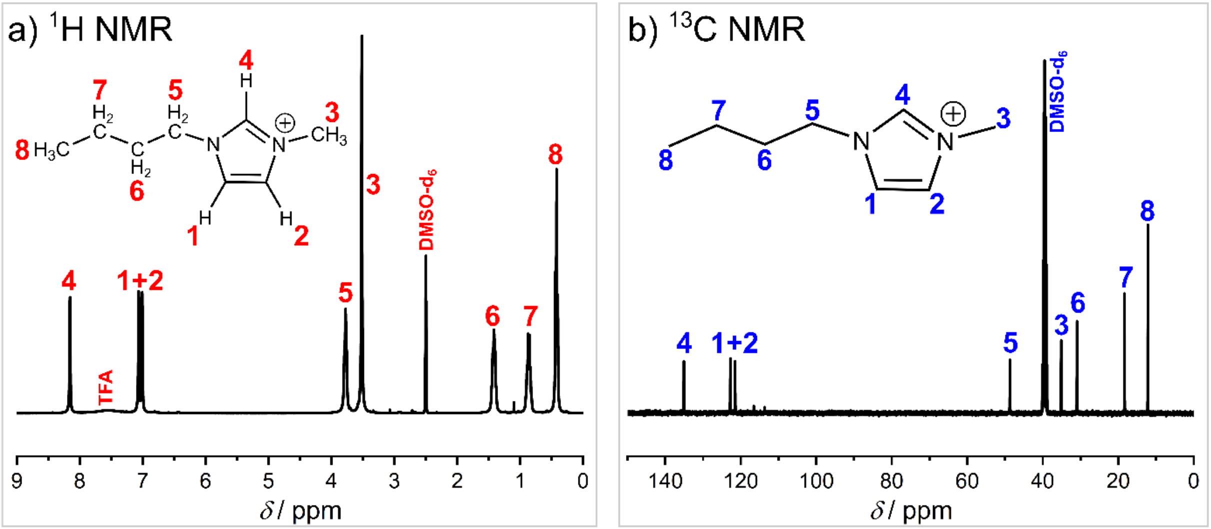

Figure S6. (a) ^{1}H NMR and (b) ^{13}C NMR spectrum of a mixture containing C$_4$mim BF$_4$ and TiCl$_4$ in a ratio of 1:0.5. The chemicals were mixed at room temperature, all spectra were measured with 400 MHz at 298 K, and a solution containing 0.1 M TFA in DMSO-d$_6$ was used as external standard.

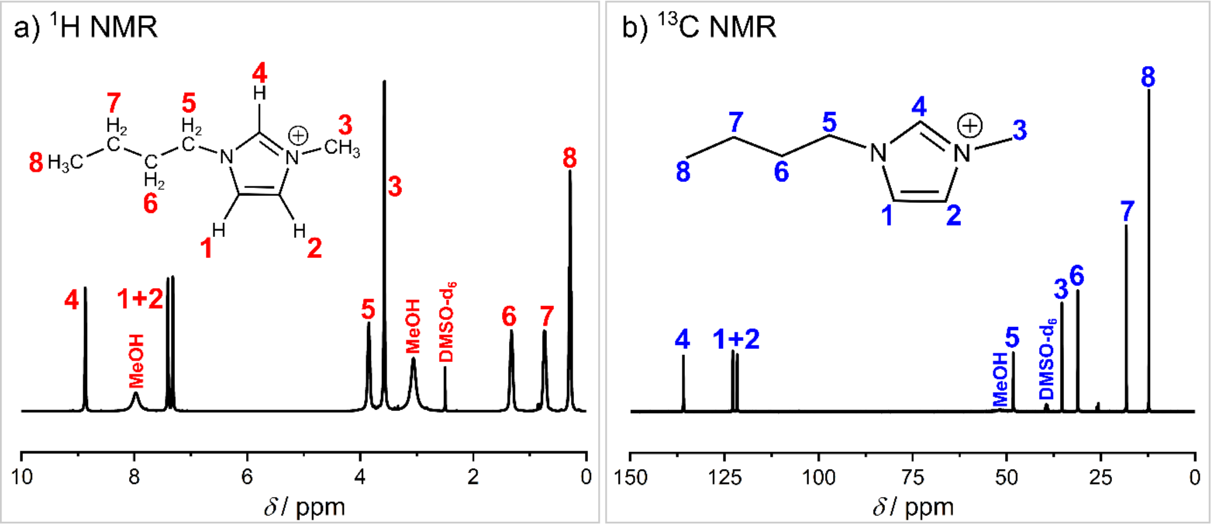

Figure S7. (a) ^{1}H NMR and (b) ^{13}C NMR spectrum of a mixture containing C$_4$mim F (with 5 %$_w$ MeOH) and TiCl$_4$ in a ratio of approximately 1:0.5. The chemicals were mixed at room temperature, all spectra were measured with 400 MHz at 298 K, and a solution containing 0.1 M TFA in DMSO-d$_6$ was used as external standard.

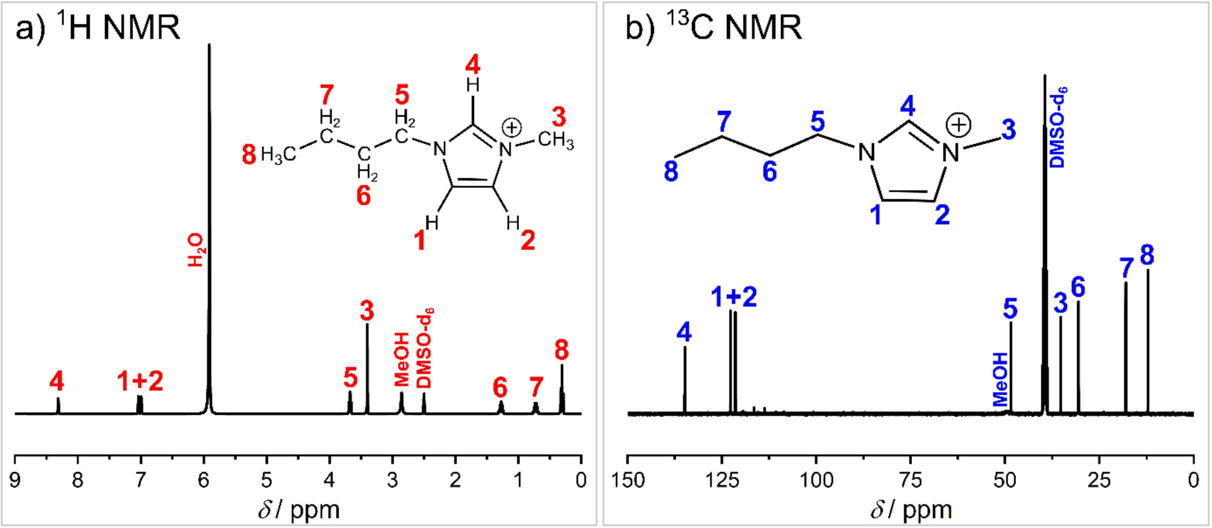

Figure S8. (a) ^{1}H NMR and (b) ^{13}C NMR spectrum of a mixture containing C_4mim F (with 5 $\%_w$ MeOH), $TiCl_4$ and H_2O in a ratio of approximately 1:0.5:6.9. The chemicals were mixed at room temperature, all spectra were measured with 400 MHz at 298 K, and a solution containing 0.1 M TFA in DMSO-d_6 was used as external standard.

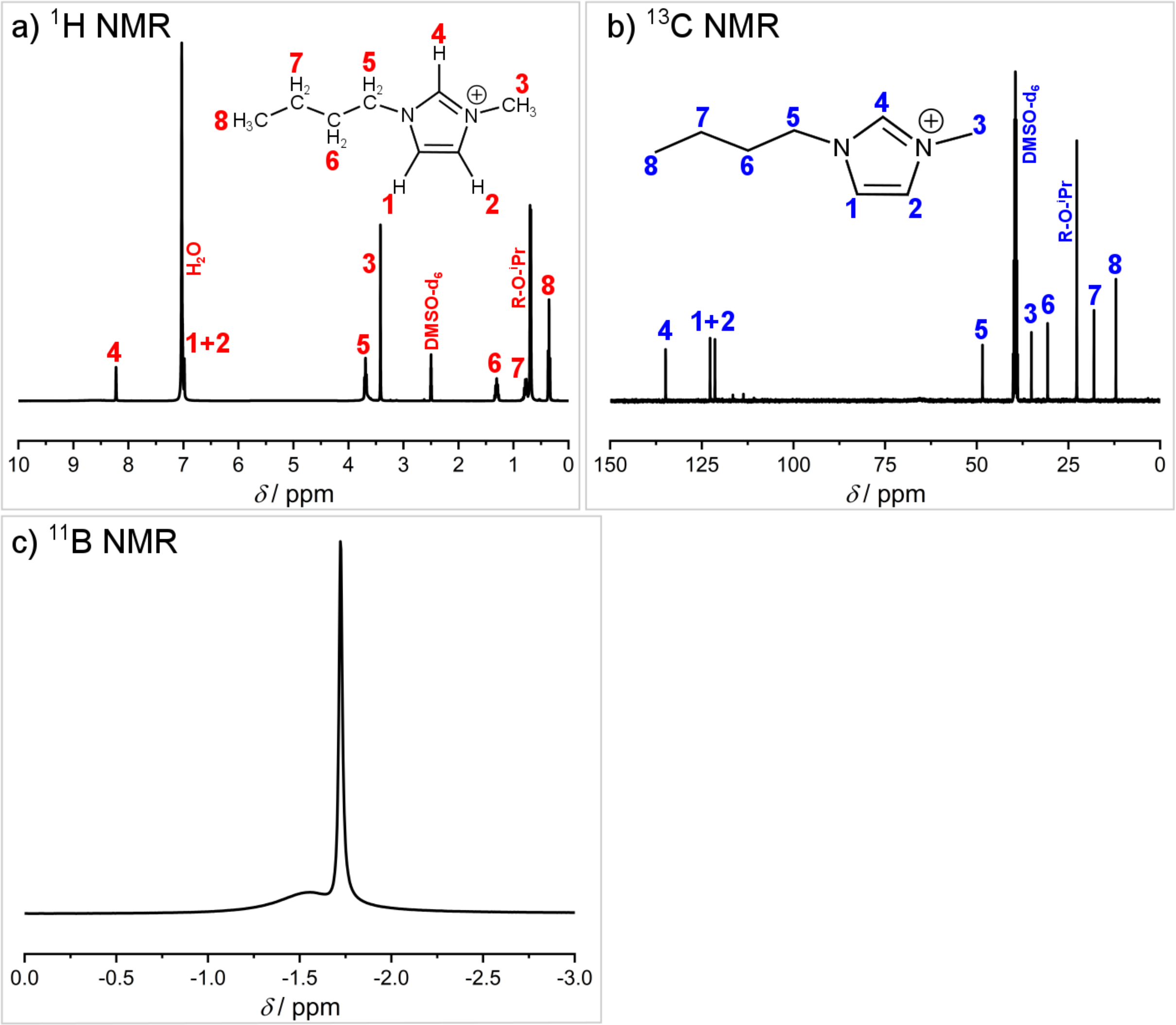

Figure S9. (a) ^{1}H NMR, (b) ^{13}C NMR and (c) ^{11}B NMR spectrum of a mixture containing C_4mim BF_4, TTIP and conc. $HCl_{aq.}$ in a molar ratio of approximately 1:0.44:2.10 (HCl):7.24 (H_2O). The chemicals were mixed at room temperature, all spectra were measured with 400 MHz at 298 K, and a solution containing 0.1 M TFA in DMSO-d_6 was used as external standard

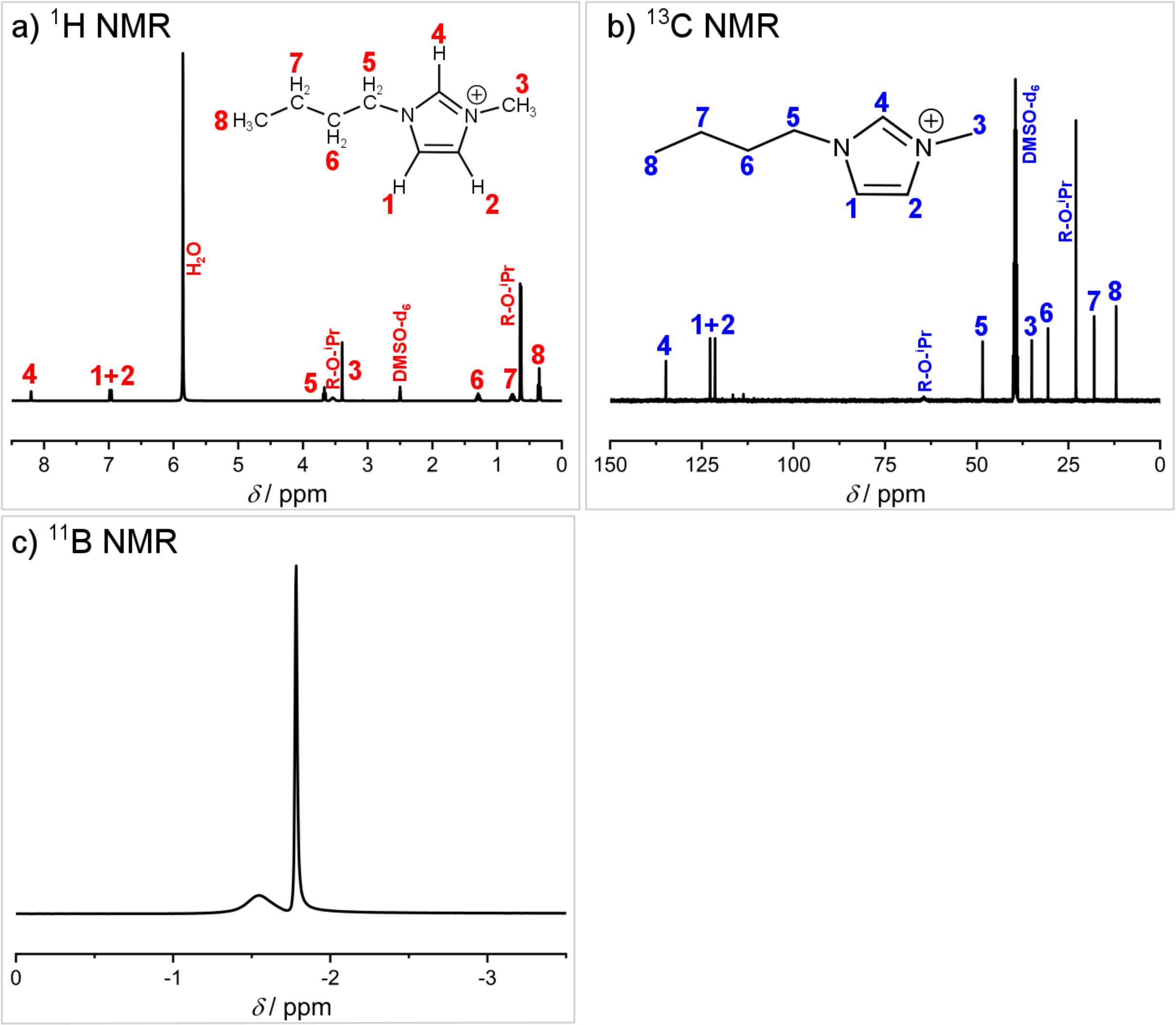

Figure S10. (a) ^{1}H NMR, (b) ^{13}C NMR and (c) ^{11}B NMR spectrum of a mixture containing C_4mim BF_4, TTIP, conc. HCl_{aq} and H_2O in a molar ratio of approximately 1:0.44:2.10 (HCl):13.74 (H_2O). The chemicals were mixed at room temperature, all spectra were measured with 400 MHz at 298 K, and a solution containing 0.1 M TFA in DMSO-d_6 was used as external standard

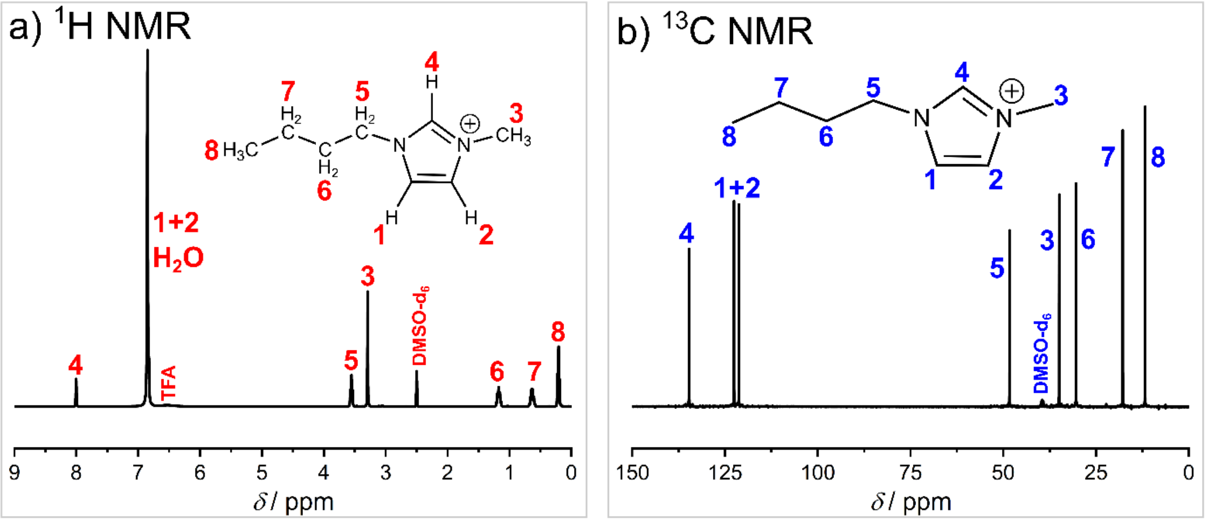

Figure S11. (a) ^{1}H NMR and (b) ^{13}C NMR spectrum of a mixture containing C_4mim BF_4, $TiCl_4$ and H_2O in a ratio of approximately 1:0.5:6.5. The chemicals were mixed at room temperature, all spectra were measured with 400 MHz at 298 K, and a solution containing 0.1 M TFA in DMSO-d_6 was used as external standard.

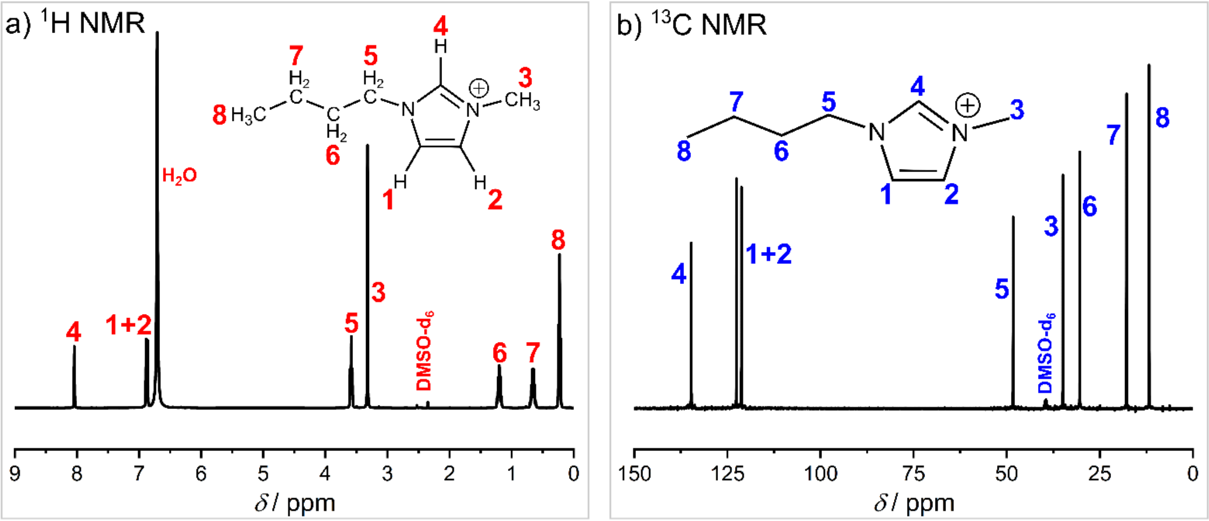

Figure S12. (a) ^{1}H NMR and (b) ^{13}C NMR spectrum of a mixture containing C_4mim BF_4, $TiCl_4$ and H_2O in a ratio of approximately 1:0.5:6.5. The solution was heated at 95 °C for 4 h and after that, the solution was cooled down for the measurements. All spectra were measured with 400 MHz at 298 K, and a solution containing 0.1 M TFA in DMSO-d_6 was used as external standard.

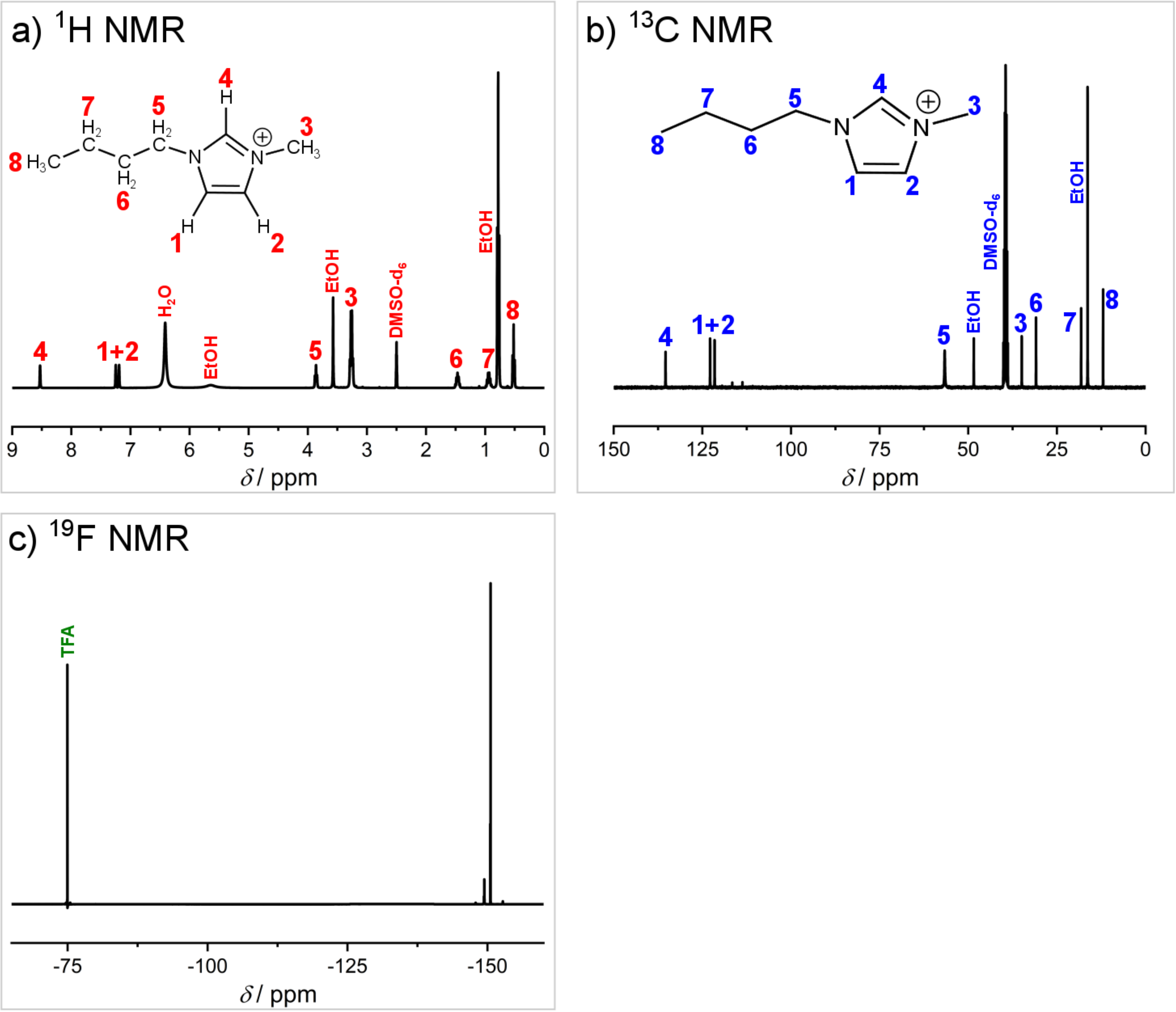

Figure S13. (a) ^{1}H NMR, (b) ^{13}C NMR and (c) ^{19}F NMR spectrum of the solution of the first washing step. All spectra were measured with 400 MHz at 298 K, and a solution containing 0.1 M TFA in DMSO-d_6 was used as external standard.

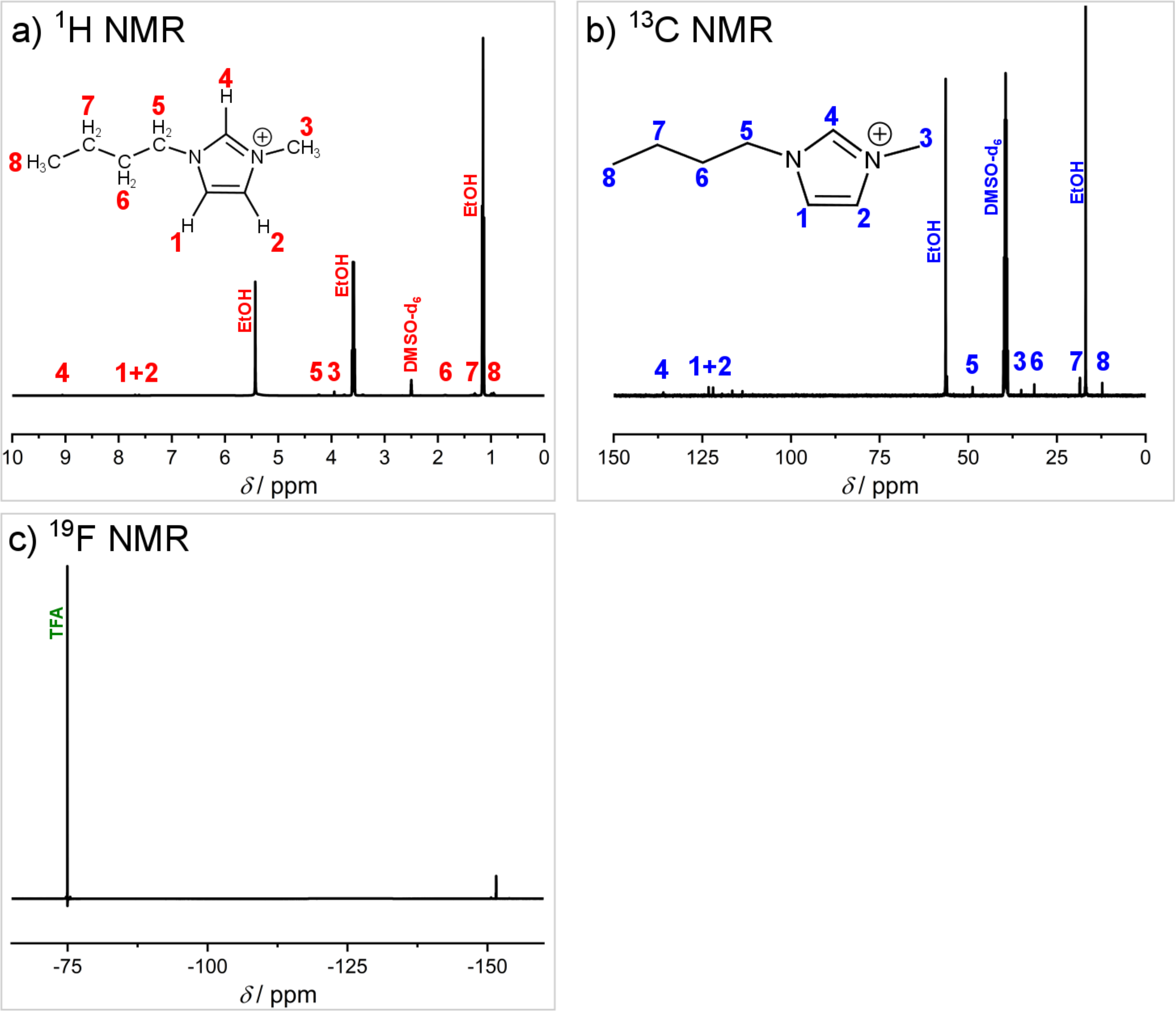

Figure S14. (a) ^{1}H NMR, (b) ^{13}C NMR and (c) ^{19}F NMR spectrum of the solution of the second washing step. All spectra were measured with 400 MHz at 298 K, and a solution containing 0.1 M TFA in DMSO-d_6 was used as external standard.

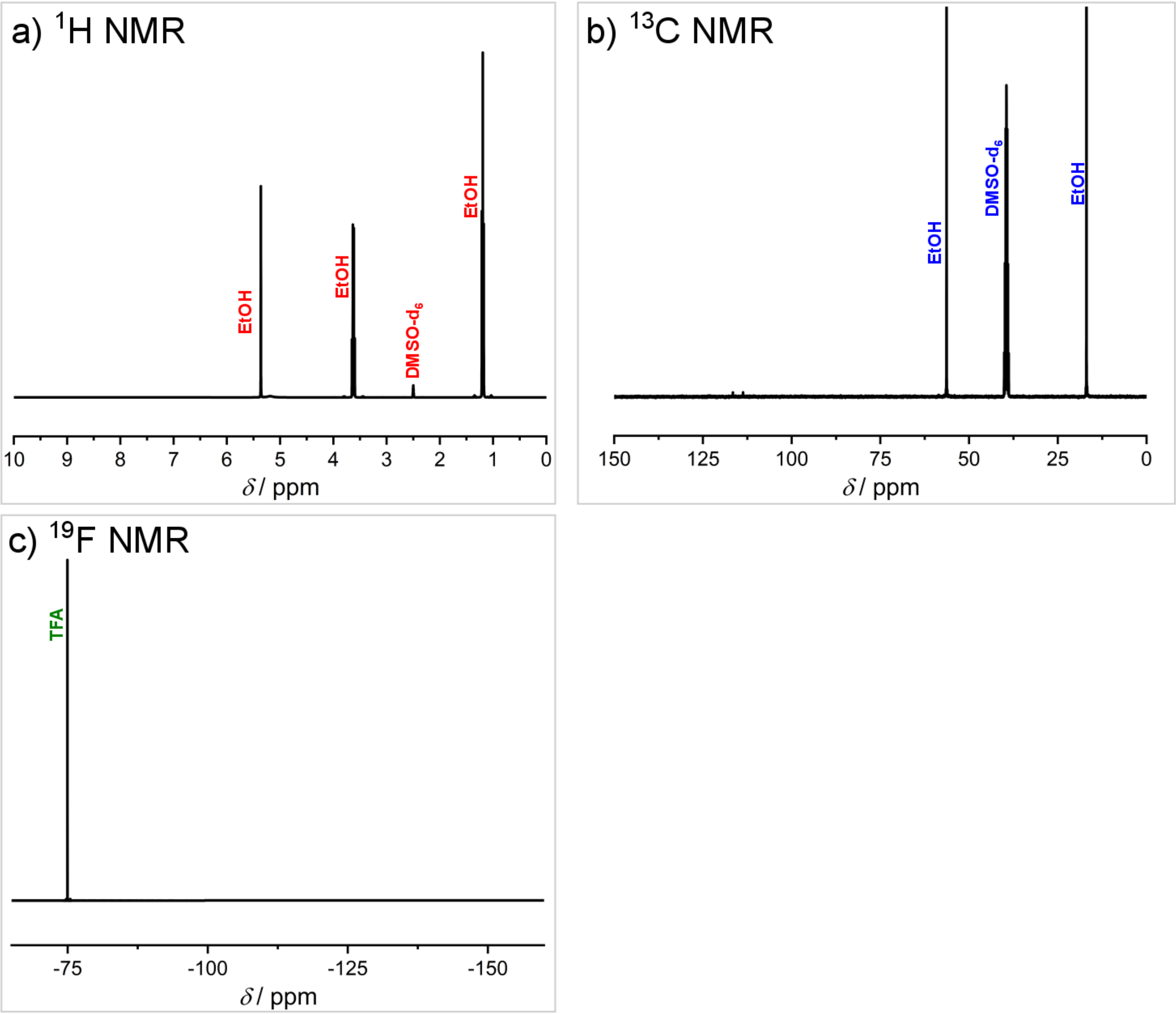

Figure S15. (a) ^{1}H NMR, (b) ^{13}C NMR and (c) ^{19}F NMR spectrum of the solution of the third washing step. All spectra were measured with 400 MHz at 298 K, and a solution containing 0.1 M TFA in DMSO-d_6 was used as external standard.

Table S2. Results of quantum chemical calculations.

Reaction	Without CPCM kJ/mol				With CPCM kJ/mol			
	Electronic energy	Gibbs free enthalpy	Total thermal energy	Total enthalpy	Electronic energy	Gibbs free enthalpy	Total thermal energy	Total enthalpy
$[TiCl_4] + BF_4^- \rightarrow [TiCl_4(BF_4)]^-$ (1)	-104,8	-53,3	-98,1	-100,5	-129,3	-77,8	-122,6	-125,0
$[TiCl_4] + 2\ BF_4^- \rightarrow [TiCl_4(BF_4)_2]^{2-}$ -cis (2)	75,4	178,2	88,7	83,7	32,3	135,1	45,6	40,6
$[TiCl_4] + 2\ BF_4^- \rightarrow [TiCl_4(BF_4)_2]^{2-}$ -trans (3)	89,7	193,0	100,3	95,4	71,8	175,1	82,4	77,5
$2\ [TiCl_4] + BF_4^- \rightarrow [(TiCl_4)_2(BF_4)]^-$ (4)	-127,0	-12,2	-112,6	-117,6	-131,5	-16,7	117,1	-122,1

xyz coordinates of the optimized structures

Titantetrachlorid $[TiCl_4]$

Ti	0.00014422457810	-0.00000000603459	-0.00017229777365
Cl	-0.00026572632064	0.00000001157206	2.17715078601122
Cl	2.05293052170329	0.00000000637895	-0.72587148724473
Cl	-1.02639900308504	1.77786237607950	-0.72555399258934
Cl	-1.02639901687571	-1.77786238799592	-0.72555400840349

Tetrafluoroborate BF_4^-

B	-0.00304780058553	0.00532335714883	-0.00219652732509
F	-0.00308958858814	0.00532115683351	1.40941813339574
F	1.32777029056695	0.00534567817772	-0.47271781014204
F	-0.66847901040832	-1.14721665730003	-0.47268663011369
F	-0.66851489098497	1.15784446513997	-0.47268216581492

$[TiCl_4(BF_4)]^-$ (1)

Ti	-0.64561424970272	-0.78709237134729	0.35520233021648
Cl	0.47352991323288	-0.67217020135451	2.28731362468206
Cl	-1.45571195719346	1.08038841371913	-0.54444015306453
F	1.11617485551081	-0.29735529021588	-0.49542892317473
B	1.90136283866269	-0.06366159140105	-1.82480575778160
F	0.97074117917147	-0.17568999080609	-2.82604038393934
Cl	-2.60507207857881	-1.33126913919143	1.36018337746862
Cl	-0.56305155442813	-2.66169590194129	-0.83814600652660
F	2.42497699766237	1.19831842983840	-1.69745962992342
F	2.85271305566291	-1.05231035730000	-1.84297447795694

$[TiCl_4(BF_4)_2]^{2-}$ -cis (2)

Ti	-0.99687976876223	-0.57256940252203	0.20284293741091
Cl	-1.03037464130221	-2.32694233254776	1.69710751186257
F	-0.36912254161119	0.56771881469755	1.83074503621729
Cl	-1.92779283739863	-1.76460789214360	-1.48697757733029
F	-2.88705156195221	-0.12507593041888	0.96171382224922
Cl	-1.17743180121290	1.44942942039359	-0.88988730844693
Cl	1.15672451611359	-0.91369508945307	-0.42019441081106
B	-4.38431181615730	-0.21877577368596	0.94302533015726
F	-4.71792949406110	-1.56054052630124	0.99790374987472
F	-4.82481116259704	0.46777401494860	2.06566396967801
F	-4.82137112206147	0.37741388813471	-0.22711835501325
B	0.69162926684732	1.18810994602033	2.69194969140695
F	1.38116667181129	2.09706198681589	1.90875770723915
F	0.02154640287448	1.81136951359998	3.73509752245327
F	1.50924288946958	0.16737636246189	3.14433537305219

$[TiCl_4(BF_4)_2]^{2-}$ -trans (3)

Ti	-0.97259209620226	-0.39194004808979	-0.24339137717700
Cl	1.02216563471011	0.22403031755203	-1.24152020764779
Cl	0.19348477276444	-1.30950794847844	1.54032518954974

F	-1.03867486364187	1.35771894017702	0.71435204261415
B	-1.11839528479266	2.83254478526430	1.09731817730782
F	-2.42715092056392	3.06009900104979	1.47060458790865
F	-0.91349388338736	-2.14416760427878	-1.19491457600395
Cl	-2.97617843885832	-1.00886605598800	0.76188504174529
Cl	-2.15398958009251	0.51916083995402	-2.01798864335114
F	-0.23854191074548	2.99022915272313	2.15095918709679
F	-0.74841721529883	3.55517695396678	-0.01325314840149
B	-0.58488441107960	-3.53008654390224	-1.74234571588503
F	-1.02737508723008	-3.52645388175239	-3.05117411574527
F	0.77945106434798	-3.68294038343815	-1.64655559414472
F	-1.27874277992965	-4.42702352475928	-0.95736384786604

$[(TiCl_4)_2(BF_4)]^-$ (4)

Ti	-0.79063535688971	-0.63197769917738	0.03603523648309
Cl	-0.83099661711233	1.12490700714127	1.37189479533634
F	-2.70869479646192	-1.67325537680077	0.81167175013780
Cl	0.14629558634080	-2.07782259253886	1.50636698510804
Cl	1.01471005546527	-0.34450692461824	-1.20739921944598
Cl	-2.27310627469703	0.14392356877778	-1.48996157530320
F	-1.57381247111642	-2.66608817321183	-0.83902029432114
B	-2.71058263830513	-2.80887152485321	-0.02391195095272
F	-3.89373409208140	-2.89190344695489	-0.78230072109222
F	-2.67098135916065	-4.00189769239361	0.72415642563630
Ti	-4.65079846925339	-4.96498181488944	-0.02257869584949
Cl	-5.47941366375708	-3.63452750931242	1.61248646634409
Cl	-6.52090503848701	-5.12315826450747	-1.18937923574521
Cl	-4.57593823830957	-6.82493153602869	1.16639972905949
Cl	-3.25892358617439	-5.64806440063222	-1.67452073539519

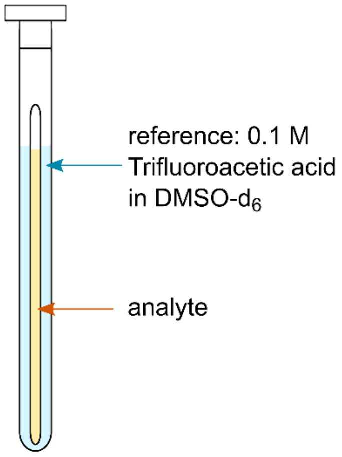

Figure S16. Schematic illustration of the NMR capillary setup.
